# 16S rDNA Full-Length Assembly Sequencing Technology Analysis of Intestinal Microbiome in Polycystic Ovary Syndrome

**DOI:** 10.3389/fcimb.2021.634981

**Published:** 2021-05-10

**Authors:** Sitong Dong, Jiao jiao, Shuangshuo Jia, Gaoyu Li, Wei Zhang, Kai Yang, Zhen Wang, Chao Liu, Da Li, Xiuxia Wang

**Affiliations:** ^1^ Center of Reproductive Medicine, Shengjing Hospital of China Medical University, Shenyang, China; ^2^ Department of Orthopedic Surgery, Shengjing Hospital of China Medical University, Shenyang, China; ^3^ Department of Research and Development, Germountx Company, Beijing, China; ^4^ Department of Biological Information, Kangwei Medical Analysis Laboratory, Shenyang, China

**Keywords:** PCOS (polycystic ovarian syndrome), 16S-FAST, intestinal microbiome, insulin resistance, overweight, enterotype

## Abstract

**Objective:**

To study the characteristics and relationship of the gut microbiota in patients with polycystic ovary syndrome (PCOS).

**Method:**

We recruited 45 patients with PCOS and 37 healthy women from the Reproductive Department of Shengjing Hospital. We recorded their clinical indexes, and sequenced their fecal samples by 16S rDNA full-length assembly sequencing technology (16S-FAST).

**Result:**

We found decreased α diversity and different abundances of a series of microbial species in patients with PCOS compared to healthy controls. We found LH and AMH were significantly increased in PCOS with *Prevotella* enterotype when compared to control women with *Prevotella* enterotype, while glucose and lipid metabolism level remained no significant difference, and situations were opposite in PCOS and control women with *Bacteroides* enterotype. *Ruminococcus gnavus, Prevotella stercorea, Dialister succinatiphilus* and *Bacteroides fragilis* were more abundant while *Christensenellaceae* spp. were less abundant in the PCOS group. *P. stercorea* was significantly more prevalent in PCOS-not insulin resistance (NIR) compared to control-NIR and PCOS-not overweight (NOW) patient groups compared to control-NOW groups. Kyoto Encyclopedia Genes and Genomes reflecting pathways related to lipopolysaccharide biosynthesis were more abundant in the PCOS group.

**Conclusion:**

Our study found gut microbiota that had different abundance in patients with PCOS compared to healthy controls. An intimate relationship was shown between the gut microbiota and pathological changes in PCOS. We suggest the gut microbiota should be taken into consideration in the treatment of symptoms of PCOS *via* drugs and diet.

## Introduction

Polycystic ovary syndrome (PCOS) is a reproductive endocrine disorder affecting 5%–10% of women of reproductive age (Aversa et al., 2020). The pathophysiological mechanism of PCOS is currently unclear. It is often characterized by polycystic ovarian changes, oligomenorrhea, and elevated androgen levels. Some patients can have metabolic syndrome with various clinical manifestations accompanied by obesity, type 2 diabetes, hyperlipidemia, insulin resistance hypertension, and high-risk cardiac factors for cerebrovascular disease. Some patients also have an increased risk of infertility, miscarriage, complications during pregnancy, and endometrial cancer (Escobar-Morreale, 2018).

Ten trillion bacteria exist within the human intestine, with the total number of bacterial genes about 150 times more than the number of human genes (Kamdar et al., 2013). The intimate exchange of information between intestinal microbes and the host plays an important role in the regulation of metabolism, immunity, and the nerve system (Rangan and Hang, 2017). The composition of the microbiome is influenced by age, ethnicity, diet, and medications, which, in turn, affects a host’s metabolism and immune response (McPhee and Schertzer, 2015). Emerging evidence shows many metabolic diseases, such as type 2 diabetes, nonalcoholic fatty liver, insulin resistance, and cardiovascular diseases (Qin et al., 2012; Qin et al., 2014; Jie et al., 2017; Woodhouse et al., 2018; Pierantonelli and Svegliati-Baroni, 2019), are related to changes in the gut microbiota spectrum.

Recently, many studies have used 16S ribosomal (r)RNA to compare the intestinal microbiome of patients with PCOS and healthy people (**Supplementary Table 1**). Alpha diversity mainly focuses on the number of species in a local uniform habitat, and, therefore, is also called habitat-within-habitat diversity. Patients with PCOS have a decreased α diversity compared to healthy people (Lindheim et al., 2017; Insenser et al., 2018; Torres et al., 2018), while some studies showed no difference (Qi et al., 2019; Zeng et al., 2019). Beta diversity refers to the difference in species composition between different habitat communities along the environmental gradient, which is also called between-habitat diversity. Studies have shown that patients with PCOS had a different β diversity from healthy people (Lindheim et al., 2017; Insenser et al., 2018; Torres et al., 2018; Qi et al., 2019; Zeng et al., 2019). *Bacteroides* is a major genus in intestinal flora. Several species of *Bacteroides* are increased or decreased in number in patients with PCOS compared to healthy people in studies by Terros et al., Zeng et al., Qi et al., Liu et al., Zhang et al., and Chu et al (Liu et al., 2017; Torres et al., 2018; Qi et al., 2019; Zeng et al., 2019; Zhang et al., 2019; Chu et al., 2020). Co-abundance groups (CAG) refers to a cluster of bacterial species based on their relative abundance. A study by Liu et al. showed that some CAGs that are altered in PCOS correlated significantly with inflammation, hyperinsulinemia, hyperandrogenism, and obesity (Liu et al., 2017). Insulin resistance and inflammation had a positive correlation with the abundance of bacteria belonging to the *Bacteroidaceae* family, which is increased in PCOS, while the testosterone level had a negative correlation with the abundance of *Prevotellaceae*, a genus found decreased in PCOS in the study of Zeng et al. (2019). Short-chain fatty acids (SCFAs) are substances released by bacteria in the human body when digesting fiber in the intestine. Two good metagenomic species (MGS) that were decreased in patients with PCOS, *Faecalibacterium prausnitzii* and *Bifidobacterium* spp., showed a positive correlation with SCFAs (Zhang et al., 2019). What is more, when Qi et al. transplanted the intestinal bacteria of patients with PCOS into mice, the mice developed a PCOS-like phenotype with a decrease in the gut bacteria bile acids, glycodeoxycholic acid (GDCA), and tauroursodeoxycholic acid (TUDCA), and in the intestinal immune factor, interleukin (IL)-22. After giving the PCOS-like mice GDCA or interleukin (IL)-22 treatment, hormone abnormalities, estrous cycle disorders, polycystic ovaries, decreased fertility, and insulin resistance were significantly improved (Qi et al., 2019).

The term “16S” refers to 16S rDNA (or 16S rRNA). The 16S rDNA is a component of the small prokaryotic ribosomal subunit. The gene that encodes 16S rDNA has a length of about 1542 bp, including nine variable regions and 10 conserved regions. The sequence of conserved regions reflects an inter-species genetic relationship, while the variable region sequence can reflect differences between species. Traditional 16S rDNA segment sequencing technology only detects the several 16S hypervariable regions of bacteria, with the detection level only reaching the genus level. 16S rDNA full-length assembly sequencing technology (16S-FAST) can sequence full-length 16S, which provides more information than some segments, and can perform further classifications to reach a species level (Karst et al., 2018). Therefore, we used 16S-FAST to analyze differences in the intestinal microbiome of patients with polycystic ovary syndrome and healthy women in northeast China for the first time. On this basis, a correlation analysis of microbiome abundance and clinical indicators as well as enterotype was carried out. After analyzing the gene function composition of a sequenced microbial genome, we used the species composition obtained by 16S-FAST to infer the composition of functional genes in a given sample, and analyzed functional differences in the bacterial population. Our work advances the elucidation of metabolic abnormalities related to symptoms of PCOS, and will contribute to the notion of having to adjust the makeup of the intestinal microbiome to improve the metabolism of patients with this disease.

## Materials And Methods

### Participants

Study participants were made up of a female population aged 18–40 (age was calculated according to the difference between screening survey and birth dates) who visited the outpatient clinic of the Reproductive Department of Shengjing Hospital, China Medical University. Patients were diagnosed with PCOS according to 2003 Rotterdam criteria (Group, 2004). The inclusion criteria for participants in the control group were regular menstrual cycles, normal ovarian morphology, and normal hormone levels. Exclusion criteria were: endocrine disorders such as diabetes mellitus, impaired glucose tolerance, hyperprolactinemia, Cushing syndrome, 21-hydroxylase deficiency, thyroid disease, androgen-secreting tumors, congenital adrenal hyperplasia, and hyperprolactinemia or other causes of hyperandrogenemia, or ovulation dysfunction; on antibiotics and hormone medications within 6 months; smokers or alcoholics; other diseases or on medication within 6 months that was known to influence the composition of the intestinal flora, and other reasons that were considered not suitable for this study. Women who were pregnant or who had been breastfeeding in the past year were also excluded. In total, 82 women returned a fecal sample, in which 37 were non-POCS controls and 45 were PCOS patients. Twenty-five women in the control group were not overweight (NOW) (body mass index [BMI] 18.5–23.9 kg/m^2^), and 12 were overweight or obese (OW) (BMI ≥24 kg/m^2^). Women with PCOS consisted of 14 NOW and 31 OW. Seventy-one women had their venous blood sample tested in routine clinical testing, of which 29 were controls and 42 were patients with PCOS. Twenty-two women did not have, and seven had, insulin resistance (IR) in the control group. Fifteen had PCOS without IR and 27 had PCOS with IR. This study protocol was approved by the Ethics Committee of Shengjing Hospital affiliated to China Medical University.

### Sampling

Fecal samples were collected in the morning during each non-menstrual period. Venous blood samples were collected in the morning after overnight fasting (≥8 h) on the 2nd to 4th day of a spontaneous menstrual cycle, or after progestin-withdrawal bleeding. Height, weight, waist and hip circumferences were recorded on the day of fecal sampling.

### Parameter Measurements

Total protein (TP), albumin (ALB), albumin/globulin (A/G), aspartate aminotransferase (AST), alanine aminotransferase (ALT), γ-glutamyltransferase (GGT), alkaline phosphatase (ALP), prealbumin (PALB), cholinesterase (CHE), total bilirubin (TBIL), direct bilirubin (DBIL), indirect bilirubin (IDBIL), total bile acid (TBA), monoamine oxidase (MAO), total cholesterol (CHOL), triglycerides (TG), high density lipoprotein cholesterol (HDL-C), low density lipoprotein cholesterol (LDL-C), apolipoprotein A1 (apoA1), apolipoprotein B (apoB), small dense low density lipoprotein cholesterol (sd-LDL), fasting plasma glucose (FPG), sex hormone-binding globulin (SHBG) and 25-hydroxy vitamin D determination (25-(OH)VitD) were measured by enzyme-linked immunosorbent assay. Follicle-stimulating hormone (FSH), luteinizing hormone (LH), estradiol (E_2_), total testosterone (TT), prolactin (PRL), progestin (Prog), free triiodothyronine 3 (FT3), free triiodothyronine 4 (FT4), thyroid stimulating hormone (TSH), and fasting plasma insulin (FINS) were measured by chemiluminescence immunoassay. Anti-Müllerian hormone (AMH) was measured by enzyme immunoassay. The waist-to-hip ratio (WHR) was calculated as a waist circumference divided by a hip circumference. BMI was calculated as weight (kg) divided by height (m) squared. Free Androgen Index (FAI) was calculated as 100×TT (ng/mL)×3.467/SHBG (nmol/L)and Homeostatic Model Assessment for Insulin Resistance (HOMA-IR) was calculated as FPG (mM) × FINS (mIU/L)/22.5.

### 16s Full-Length Library Construction Technology

Patients were instructed to collect fecal samples into fecal DNA storage tubes (CW2654, CwBiotech, Beijing, China) and then the samples were stored and sent to laboratory under room temperature. Bacterial DNA was extracted using an intestinal DNA extraction kit (Qiagen Fecal DNA Extraction Kit, Qiagen, Hilden, Germany). Quantitative and qualitative analysis, and quality control of the extracted DNA was subsequently performed. For samples that passed quality control, 10 ng DNA was used to construct the following full-length 16S library. Full-length PCR amplification system 1 was configured, and 10 ng of DNA template was added to perform the first round of three cycles of full-length amplification. The amplified product was purified by magnetic beads.

Full-length PCR amplification system 2 was nest configured, and the second round of a 32-cycle full-length amplification was performed on the product of the first round of magnetic bead purification. The full-length amplified product was then purified by magnetic beads, and the purified product was analyzed quantitatively and qualitatively.

We then constructed a splicing library. Ten nanograms of full-length amplification was taken for transposase digestion. A spliced library PCR amplification system 1 was configured. Ten microlitres of the digested product was used for PCR amplification. A DNA fragment was selected from PCR-amplified products. A spliced library PCR amplification system 2 was then configured, and 2 μL of the product, after magnetic bead screening and purification, was used for the second round of a spliced library PCR amplification. The amplified product was also purified by magnetic beads. The product of this round was the spliced library.

We also constructed a link library. A ligation library PCR amplification system 1 was constructed. Three nanograms was used for the first round of eight cycles of PCR amplification. The amplified product was purified by magnetic beads. Ligation library PCR amplification system 2 was configured, and 2 μL of the product of the first round of library construction and purification was used to perform the second round of eight cycles of PCR amplification. The PCR amplification product was purified by magnetic beads. The product of this round was known as the ligation library. Electrophoresis and the mesurements of Qubit concentrations were performed on spliced and ligated libraries. A library that passed quality control was sent for sequencing.

### Bioinformatics Analysis

Unique Molecular Identifier (UMI) pairing relationships were extracted through the ligation library. All sequences corresponding to each paired UMI from the spliced library were extracted. For each paired UMI sequence, Cutadapt V1.2.1 was used to excise primers and UMI tags, and every UMI was assembled to a full-length 16S sequence through default parameters using software SPAdes V3.13.1. Mothur V1.42.0 and SILVA_132_SSURef_Nr99 databases were used for all the above sequences to perform species annotations with default parameters. The α bacterial diversity of the gut microbiota community was estimated by qiime1 V1.8.0. The difference between groups was calculated by a Mann–Whitney *U* test using a scipy 1.3.1 package in python3.6. The β diversity was analyzed by an R3.6.1 package vegan2.5-3 analysis. The *P* value from partial least squares-discriminate analysis (PLS-DA) was calculated by matching the Adonis method. The Linear discriminant analysis Effect Size (LEfSe) was conducted by LEfSe version 1.0, parameter setting at 2, to discover gene or functional characteristics that could best explain differences between the groups. Cytoscape_v3.8.0 was used to construct a network diagram, and Spearman in SparCC was used to calculate correlation and *P* values. For the analysis of genus level and above, results with a correlation greater than 0.5 and *P* value ≤0.05 were retained; for species level, results with a correlation greater than 0.6 and *P* value ≤0.05 were retained. Mann–Whitney *U* and Kruskal–Wallis sum-rank tests of non-parametric coefficients were used to detect significant differences in clinical indexes between groups. The correlation between bacterial species and metabolic indicators was conducted by R3.6.1 package psych v1.8.4, using Spearman’s method. Heatmaps were drawn by an R package heatmap 1.0.12. PICRUSt2 was used to infer the composition of functional genes in the sample, and analyze the functional differences between different samples and groups. Python3.6 package sklearn0.23.2 was used to obtain the top 10 contribution species through recursive feature elimination, and a random forest was constructed to classify and predict samples based on flora or clinical indicators. Enterotypes were determined as follows: clustering was performed using the Center Point Partition Algorithm according to the Jensen–Shannon distance between samples and the optimal number of classifications was determined by the Calinski–Harabasz (CH) index. The cluster group with the highest CH index was considered the optimal number of groups.

## Results

### Clinical Parameters Characteristic of Participants

According to the following basic information, which included weight, BMI, waistline, hipline and WHR were significantly higher in the PCOS group (*P* < 0.05). As for biochemical indices, TP, ALB, AST, ALT, GGT, ALP, PALB, CHR, MAO, CHOL, TG, apoB, and sd-LDL were higher, while HDL-C and DBIL were lower in the PCOS group (*P* < 0.05). PRL, SHBG, and FSH were lower, while FAI, TT, LH, FT3, FINS, AMH, and IR were higher in the PCOS group (**Supplementary Table 2**). A comparison of indexes between PCOS-NIR and control-NIR, PCOS-IR and control-IR, PCOS-NOW and control-NOW, and PCOS-OW and control-OW are also shown in **Supplementary Table 2**. A comparison of indexes between *Bacteroides*-dominated enterotype PCOS women and control women, *Prevotella*-dominated enterotype PCOS women and control women are shown in **Table 1**.

**Table 1:** Clinical parameters of PCOS participants classified by enterotypes.

	Control		PCOS	
	Bacteroides enterotype	Prevotella enterotype	Bacteroides enterotype	Prevotella enterotype
Age(year)	31(28-35)	31(28-32)	30(27-33)	28.5(25.75-35.25)
Height(m)	161(159-165)	163(160-172)	163(160-165)	163(158-167.5)
Weight(m)	57.75(53.25-65.3)	60(57-75)	75(62.5-83)*	68.75(56.5-80.625)
BMI(kg/m2)	22.19(19.90-25.01)	22.83(22.03-25.35)	26.95 (23.43-31.43)*	25.69 (22.33-30.30)
Waistline(cm)	75.5(72.75-82)	83(76-90)	88(79.5-101.25)*	90.5(77.5-97.5)
Hipline(cm)	94.5(90.75-100)	98(93-101)	100(93.75-107.75)*	97.5(93-110)
WHR	0.81(0.78-0.86)	0.84(0.81-0.90)	0.87 (0.83-0.92)*	0.86 (0.83-0.94)
TP(g/L)	74.5(71.6-76.8)	73.05(69.95-75.95)	77.9(74.9-80.85)*	76.2(72.55-78.225)
ALB(g/L)	46.3(44.2-48.2)	44.4(41-48.525)	47.5(44.7-50.1)	46.6(45.3-50.375)
A/G	1.66(1.54-1.81)	1.58(1.39-1.71)	1.57(1.43-1.72)	1.625(1.555-1.88)
AST(U/L)	14.2(11-17)	17(16-20)	19.5(16.25-24.5)*	18.8(15.75-23.75)
ALT(U/L)	12(8.5-14.5)	14(12.25-16.5)	21.5(14.5-37)*	17.65(11.5-40.5)
GGT(U/L)	12(10.5-17.1)	13(10.5-20.25)	24.5(16-37.5)*	21.5(17.5-35.5)
ALP(U/L)	64.6(57.85-71.3)	66.7(56.87-82.52)	82.4(64.65-98.32)*	74.8(66.72-82.87)
PALB(g/L)	0.253(0.24-0.2775)	0.26(0.17-0.29)	0.28(0.25-0.31)	0.265(0.23-0.3)
CHE(U/L)	7521(6539.5-9058.5)	7849(6683.25-8602.25)	9476(7853-10537)*	8738.5(8268-9454.25)
TBIL(umol/L)	10.8(9.45-14.85)	13.6(10.45-18.23)	9.9(8.4-12.375)	10.2(9.675-16.175)
DBIL(umol/L)	3.3(2.5-3.975)	3.75(2.43-4.58)	2.7(1.95-3.3)	2.6(2.05-3.425)
IDBIL(umol/L)	8.25(6.75-10.6)	8.85(7.73-14.08)	7.85(6-9.5)	8.15(6.8-12.55)
TBA(umol/L)	1.98(1.38-3.07)	2.69(0.96-3.98)	2.43(1.35-3.73)	1.94(1.44-5.22)
MAO(U/L)	5.3(4.5-6.81)	5.58(4.33-7.03)	6.69(5.92-7.70)	5.75(4.17-9.06)
CHOL(mmol/L)	4.34(4.19-4.65)	5(3.99-5.46)	4.77(4.4-5.8)	4.65(4.28-5.13)
TG(mmol/L)	0.72(0.56-1.21)	0.84(0.58-1.17)	1.29(0.98-2.16)*	1.285(0.685-2.42)
HDL-C(mmol/L)	1.58(1.145-1.87)	1.3(1.17-1.5)	1.2(1.02-1.4)	1.105(0.85-1.7825)
LDL-C(mmol/L)	2.56(2.28-3.14)	3.27(2.44-3.45)	2.97(2.36-4)	2.73(2.41-3.4675)
apoA1(g/L)	1.52(1.33-1.68)	1.55(1.39-1.71)	1.40(1.28-1.56)	1.35 (1.14-1.56)
apoB(g/L)	0.68(0.61-0.86)	0.83(0.66-0.96)	0.91(0.79-1.11)*	1.01(0.80-1.18)
sd-LDL(mmol/L)	0.58(0.51-1.08)	0.77(0.67-1.13)	0.97 (0.85-1.44)*	1.02 (0.55-1.47)
FPG(mmol/L)	5.24(4.82-5.51)	5.22(4.89-5.43)	5.16(4.95-5.52)	5.3(4.77-5.88)
25(OH)-VitD(ng/mL)	13.58(10.48-18.51)	13.12(12.06-16.97)	12.97(10.03-16.49)	13.04(10.09-16.84)
Prog(ng/mL)	0.63(0.27-0.76)	0.58(0.26-0.69)	0.47(0.24-0.70)	0.44(0.20-0.50)
PRL(ng/mL)	11.83(9.42-15.36)	12.55(8.82-16.31)	7.85(6.02-13.05)	10.06(7.17-13.29)
SHBG(nmol/L)	44.9(33.8-87.4)	50.3(36.65-88.2)	17.4(12.15-35.5)*	19.15(13.5-38.55)
FAI(%)	3.4(1.83-4.67)	2.92(2.29-6.57)	13.39(6.93-22.69)*	12.67(4.06-18.65)
TT(ng/mL)	0.43(0.35-0.55)	0.44(0.37-0.69)	0.68(0.51-0.78)*	0.69(0.445-0.9)
LH(mIU/mL)	3.82(2.97-5.02)	3.42(2.66-4.74)	8.36(4.78-10.25)*	8.89(5.41-12.187)#
FSH(mIU/mL)	7.31(6.49-8.94)	7.07(6.34-8.4)	6.28(5.40-7.32)	6.52(5.5475-7.42)
E2(pg/mL)	50(37-69.5)	61(43-84)	51(34.75-72.75)	49(36-74.5)
FT3(pmol/L)	4.57(4.19-4.745)	4.61(4.095-5.2)	4.9(4.54-5.07)	4.89(4.57-5.30)
FT4(pmol/L)	12.65(11.945-13.82)	13.51(12.82-14.66)	12.80(11.77-13.44)	12.92(10.97-14.24)
TSH(uIU/mL)	2.01(1.39-2.29)	1.81(1.02-2.89)	1.71 (1.04-2.42)	1.29(1.08-3.22)
FINS(mU/L)	8.9(7.15-11.45)	7.45(6.02-9.17)	18.2(9.68-23)*	11.2(8.55-23.62)
AMH(ng/mL);	3.29(1.81-5.45)	2.57(2.19-3.23)	6.78(4.52-12.86)*	9(6.07-11.61)#
HOMA-IR	2.02(1.61-2.59)	1.65(1.37-2.22)	4.02 (2.13-5.66)*	2.55 (1.90-6.23)

PCOS, polycystic ovary syndrome.

Data are presented as Median (IQR).

WHR, waist-to-hip ratio; TP, total protein; ALB, albumin; A/G, albumin/globulin; AST, aspartate aminotransferase; ALT, alanine aminotransferase; GGT, γ-glutamyltransferase; ALP, alkaline phosphatase; PALB, prealbumin; CHE, cholinesterase; TBIL, total bilirubin; DBIL, direct bilirubin; IDBIL, indirect bilirubin; TBA, total bile acid; MAO, monoamine oxidase; CHOL, total cholesterol; TG, triglycerides; HDL-C, high density lipoprotein cholesterol; LDL-C, low density lipoprotein cholesterol; apoA1, apolipoprotein A1; apoB, apolipoprotein B; sd-LDL, small dense low-density lipoprotein–cholesterol; FPG, fasting plasma glucose; 25-(OH)VitD, 25-hydroxy vitamin D determination; Prog, progesterone; PRL, prolactin; SHBG, sex hormone–binding globulin; FAI, free androgen index; TT, total testosterone; LH, luteinizing hormone; FSH, follicle-stimulating hormone; E_2_, estradiol; FT3, free triiodothyronine 3; FT4, free triiodothyronine 4; TSH, thyroid stimulating hormone; FINS, fasting plasma insulin; AMH, anti-Müllerian hormone; HOMA-IR, homeostasis model assessment.

*PCOS with Bacteroides enterotype vs. control with Bacteroides enterotype. ^#^PCOS with Prevotella enterotype vs. control with Prevotella enterotype.

### Differences in Bacterial Diversity of Gut Microbiota

**Figure 1:**
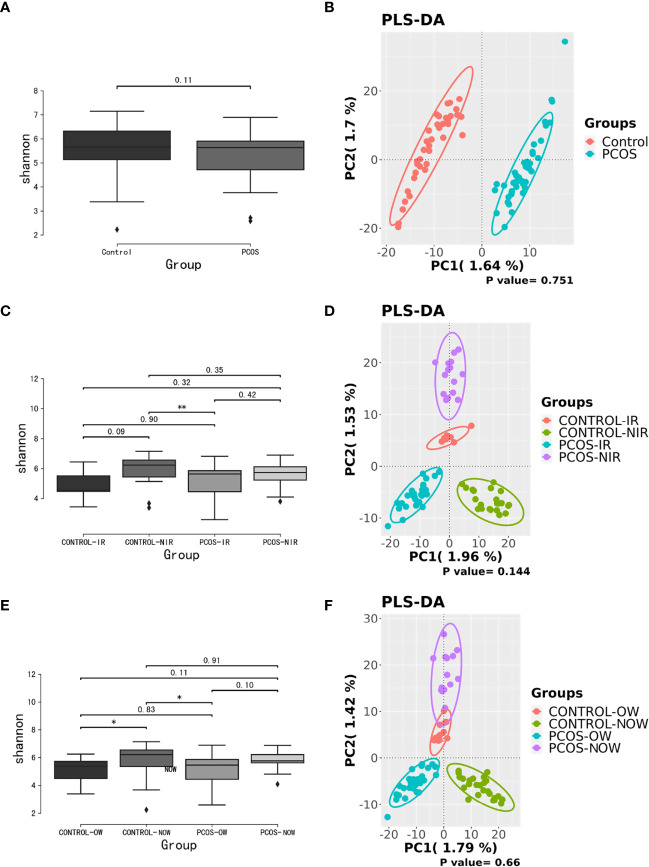
Biodiversity of the gut microbiome of participants on the ASV level. **(A)**, Alpha diversity comparison between PCOS and control groups with a box plot of the Shannon index. **(B)**, Partial least squares-discriminate analysis (PLS-DA) score plot of amplicon sequence variant (ASV) abundance in PCOS and control groups. **(C, D)**, Alpha diversity comparison and PLS-DA score plots between groups further divided by HOMA-IR. **(E, F),** Alpha diversity comparison and PLS-DA score plots between groups further devided by BMI. BMI, body mass index; HOMA-IR, homeostasis model assessment; PCOS, polycystic ovary syndrome.

Samples containing a number of contigs of more than 5000 were filtered after sequencing full length 16S rRNA. All samples met the standard. A rarefaction curve showed the abundance of species in samples with different amounts of sequencing data (**Supplementary Figure 1**). When the curve tends to be flat, it indicates that the amount of sequencing data is reasonable. We assessed α diversity by a Shannon index based on amplicon sequence variant (ASV). We observed a higher Shannon index (*P* = 0.11) in the control group, which demonstrated a decrease in α diversity in PCOS women when compared to the control group though no statistical significance was reached. Beta diversity based on ASV was assessed by PLS-DA. The outcome could not separate PCOS and control groups (*P* = 0.751; **Figure 1**).

When patients with PCOS were further classified according to HOMA-IR, the Shannon index showed a significant difference between PCOS-IR and control-NIR groups. When PCOS and control groups were both classified according to BMI, the Shannon index showed a significant difference between PCOS-OW and control-NOW groups, as well as control-OW and control-NOW groups. As for β diversity, no significant difference was observed between groups.

### Difference in Species of Bacteria Between PCOS and Control Groups

**Figure 2:**
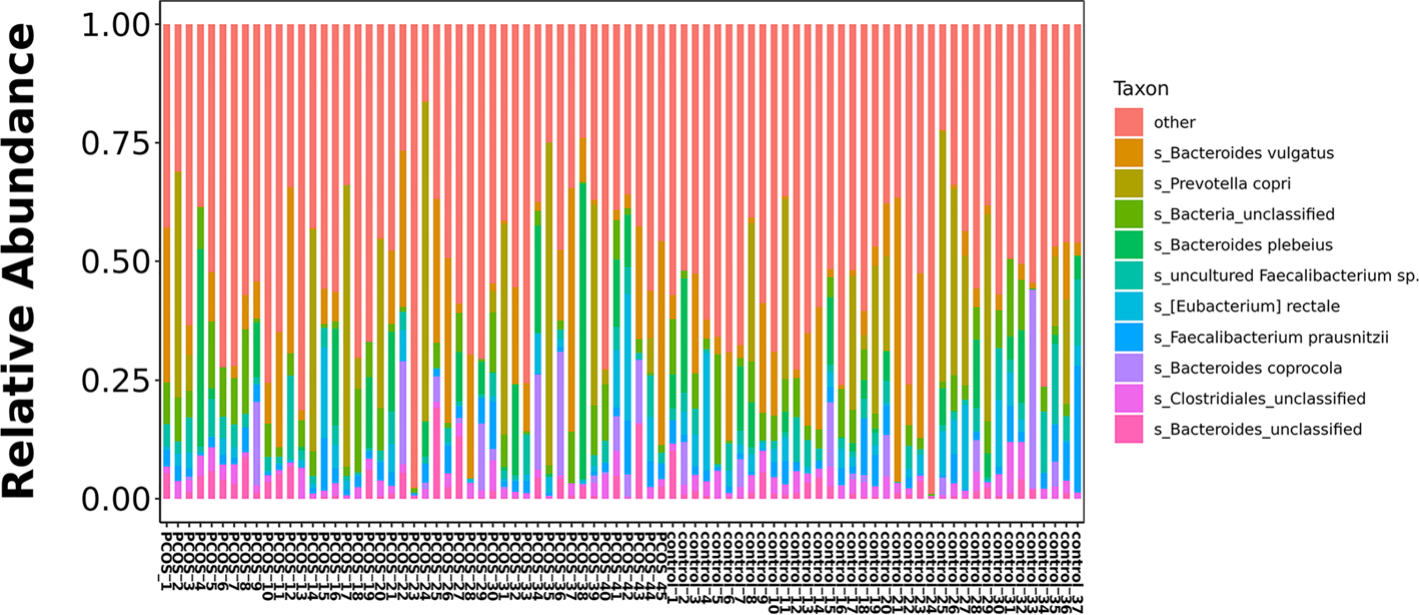
Histogram of top 10 species composition in all samples.

**Figure 3:**
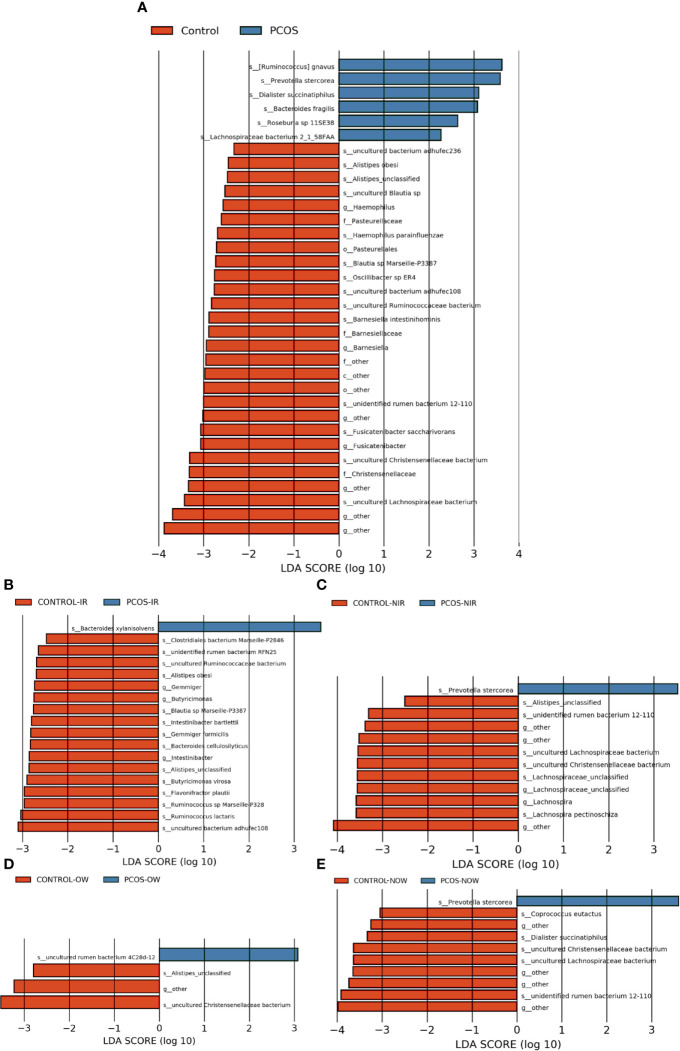
Species of different abundances between groups. **(A)**, Linear discriminant analysis effect size (LEfSe) for species with different abundances in PCOS and control groups. **(B, C)**, Species with different abundances in control-IR *vs.* PCOS-IR, and control-NIR *vs.* PCOS-NIR groups. **(D, E)**, Species with different abundances in control-OW *vs.* PCOS-OW, and control-NOW *vs*. PCOS-NOW groups. Linear discriminant analysis (LDA) over 2 was shown.

**Figure 4:**
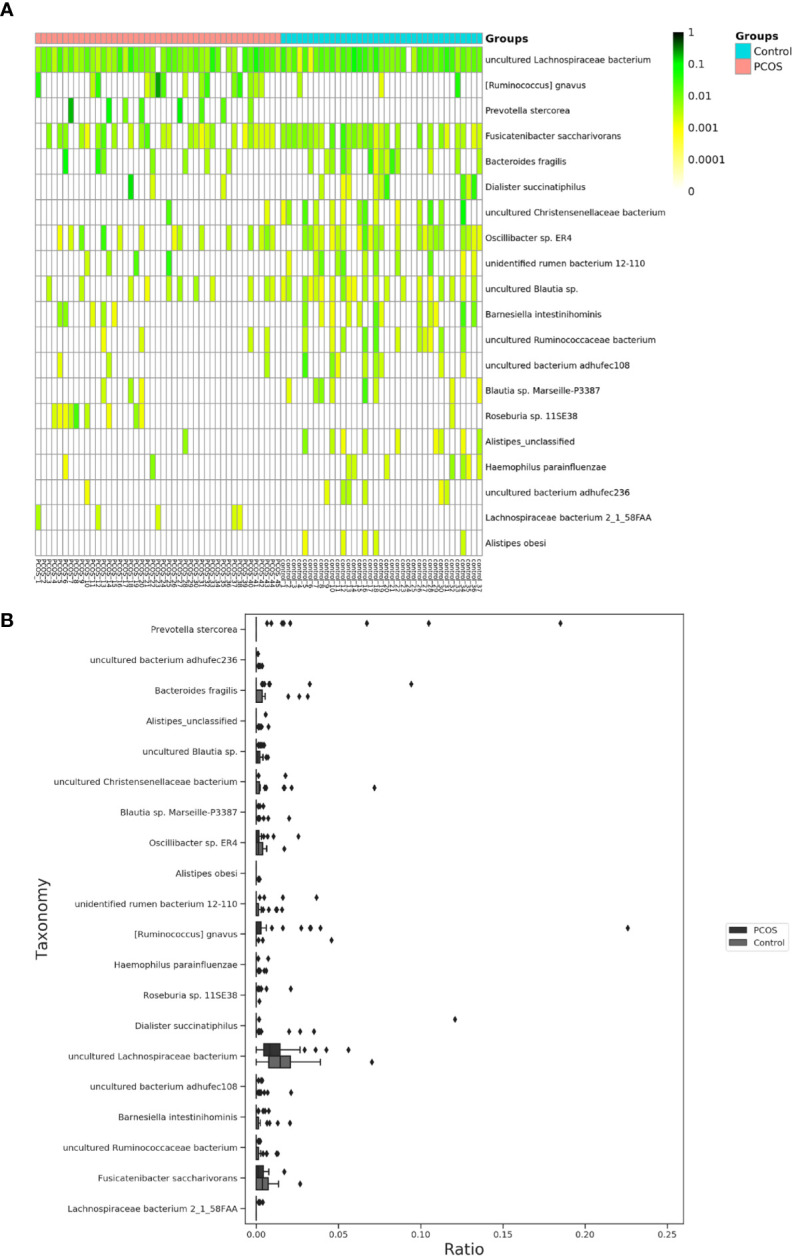
Distinct species of microbiota between PCOS and control groups. **(A)**, Heatmap of species that had different abundances. A color gradient was used to show abundance (white=not detected). The number of samples is shown on the bottom, and the name of species is shown on the right. **(B)**, Box plot of species with different abundances. The species listed on **(A, B)** were those that showed a significant difference in abundance in LEfSe analysis. LEfSe, linear discriminant analysis effect size; PCOS, polycystic ovary syndrome.

The relative abundance of the top 10 taxa at a species level (**Figure 2**) showed that *Bacteroides vulgatus* and *Prevotella copri* were the two major species. The LEfSe was then used to further investigate the microbiota in order to explain the difference between PCOS and control groups. *Ruminococcus gnavus*, *Prevotella stercorea, Dialister succinatiphilus*, *Bacteroides fragilis*, *Roseburia* spp. *11SE38*, and *Lachnospiraceae bacterium 2_1_58FAA* at a species level was more abundant in the PCOS compared to control group with a LDA score over 2. While *Christensenellaceae*, *Barnesiellaceae*, and *Pasteurellaceae* at a family level, *Fusicatenibacter*, *Barnesiella*, and *Haemophilus* at a genus level, uncultured *Lachnospiraceae* bacteria, uncultured *Christensenellacceae* bacteria, *Fusicatenibacter saccharivorans*, unidentified rumen bacterium 12-110, *Barnesiella intestinihominis*, uncultured *Ruminococcaceae* bacteria, uncultured bacterium adhufec108, *Oscillibacter* sp. *ER4*, *Blautia* sp. *Marseille-P3387*, *Haemophilus parainfluenzae*, uncultured *Blautia* sp., *Alistipes_obesi, Alistipes_*unclassified and uncultured bacterium adhufec236 at a species level were more abundant in the control compared to PCOS group. Moreover, *P. stercorea* showed a greater abundance in the PCOS-NIR compared to control-NIR group, and in the PCOS-NOW when compared to control-NOW group, respectively (**Figure 3**). The distribution of the different microbiota species in the samples is shown in **Figure 4**.

### Correlation Between Clinical Indexes and Species

**Figure 5:**
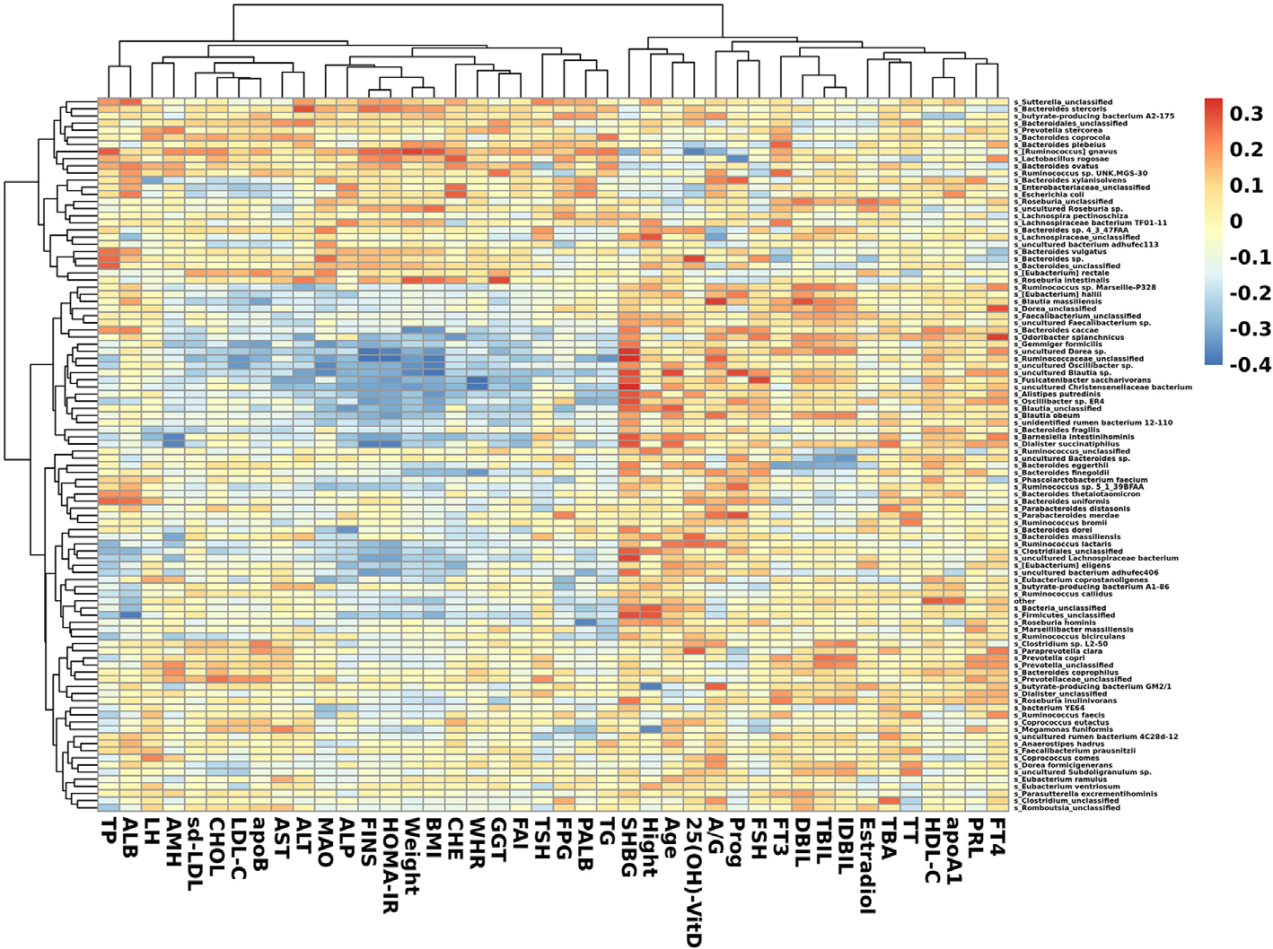
Heatmap of correlation between clinical indexes and species. Species with the top 100 abundances that were detected in samples are listed on the left, and clinical indexes are shown on the bottom. Abundances of species are shown by different colors.

In species that were significantly abundant in patients with PCOS, *R. gnavus* was positively correlated with FINS, HOMA-IR, weight, BMI, TP, and TG. *P. stercorea* showed a mild positive correlation with LH and AMH levels. While among species abundant in the control group, *F. saccharivorans*, uncultured *Christensenellacceae* bacteria, and *B. intestinihominis* correlated positively with FINS, HOMA-IR, weight, and BMI, but had a negative correlation with SHBG (**Figure 5**).

### Prediction of Alterations in Metabolic Pathways

**Figure 6:**
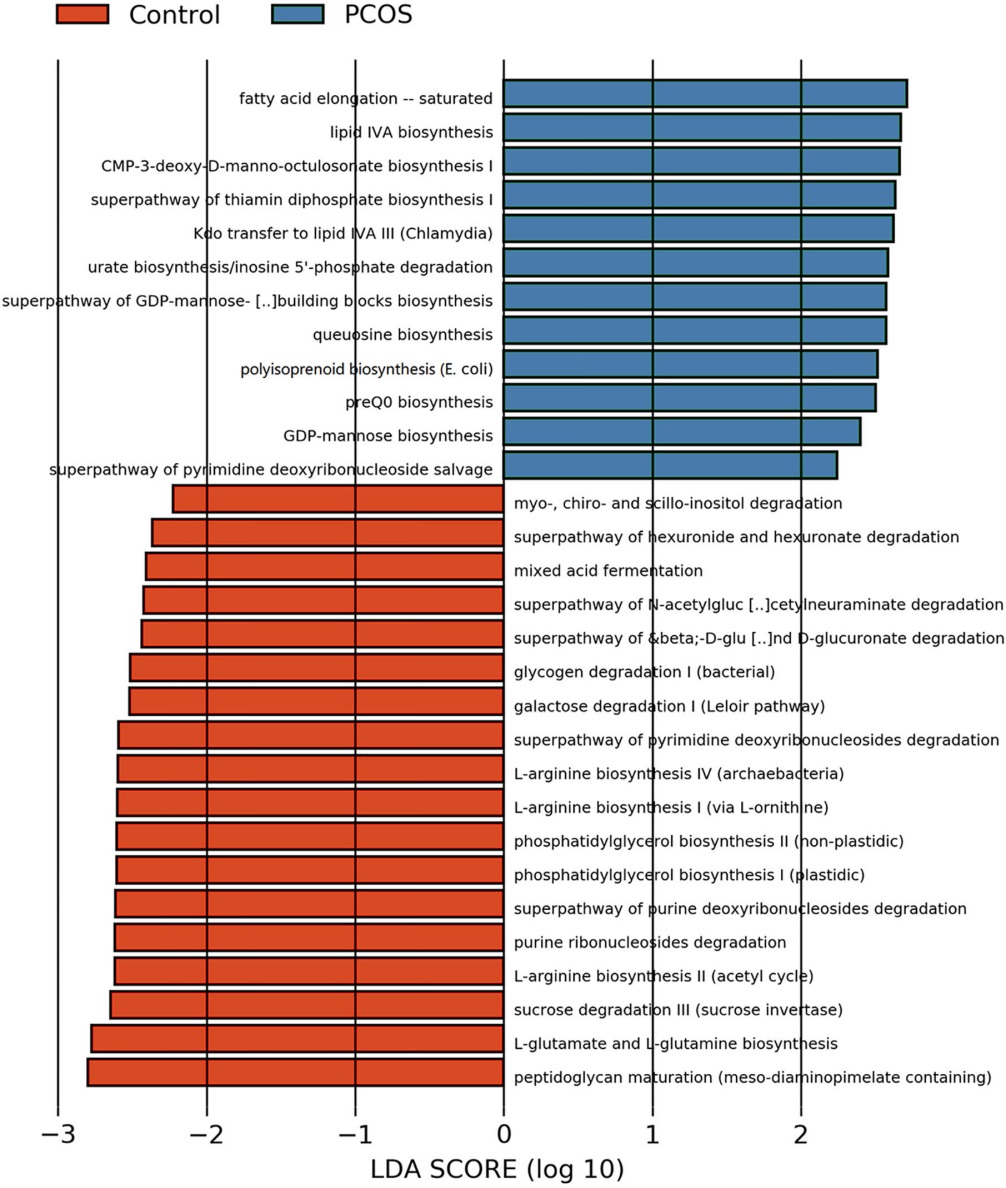
LEfSe difference analysis based on functions in pathways. LEfSe, linear discriminant analysis effect size.

After analyzing the composition of gene functions in the sequenced microbial genome, we inferred functional genes in the sample through the species composition obtained by 16S-FAST so as to analyze functional differences between different samples and groups. Functional differences on KEGG and pathway between PCOS and control groups are shown in **Figure 6** and **Supplementary Figure 2**. Fatty acid elongation – saturated, lipid IVA biosynthesis, CMP-3-deoxy-D-manno-octulosonate biosynthesis I, superpathway of thiamin diphosphate biosynthesis I, Kdo transfer to lipid IVA III (Chlamydia), urate biosynthesis/inosine 5’-phosphate degradation, superpathway of GDP-mannose-derived O-antigen building blocks biosynthesis, queuosine biosynthesis, polyisoprenoid biosynthesis (E. coli), preQ0 biosynthesis, GDP-mannose biosynthesis and superpathway of pyrimidine deoxyribonucleoside salvage were significantly abundant in PCOS, while peptidoglycan maturation (meso-diaminopimelate containing), L-glutamate and L-glutamine biosynthesis, sucrose degradation III (sucrose invertase), L-arginine biosynthesis II (acetyl cycle), purine ribonucleosides degradation, superpathway of purine deoxyribonucleosides degradation, phosphatidylglycerol biosynthesis II (non-plastidic), phosphatidylglycerol biosynthesis I (plastidic), L-arginine biosynthesis I (via L-ornithine), L-arginine biosynthesis IV (archaebacteria), superpathway of pyrimidine deoxyribonucleosides degradation, galactose degradation I (Leloir pathway), glycogen degradation I (bacterial), superpathway of &beta;-D-glucuronide and D-glucuronate degradation, superpathway of N-acetylglucosamine, N-acetylmannosamine and N-acetylneuraminate degradation, mixed acid fermentation, superpathway of hexuronide and hexuronate degradation and myo-, chiro- and scillo-inositol degradation were more abundant in control group with significant difference.

### Network of Microbiota in Different Groups

**Figure 7:**
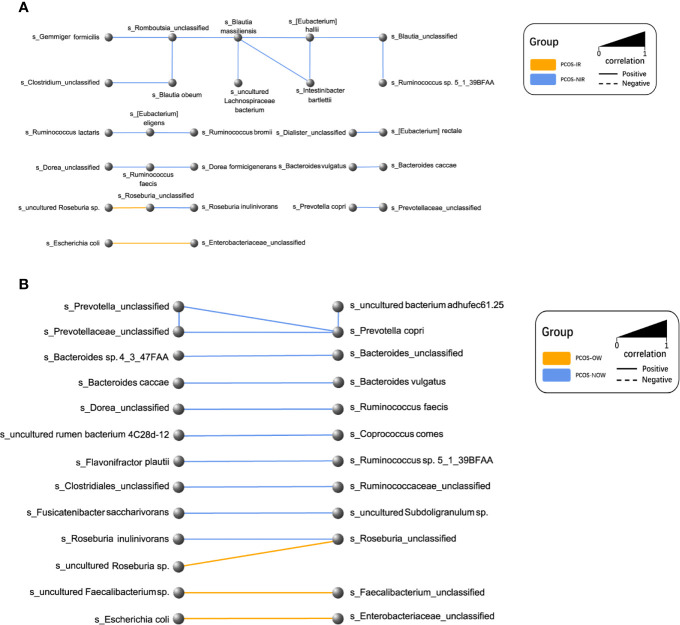
Network plot of species. Every node represented a species. Two species that had a positive relationship were connected with a solid line, and a negative relationship with a dashed line. The width of the line represented the strength of association. **(A)** Relationships between PCOS-IR and PCOS-NIR. **(B)** Relationships between PCOSOW and PCOS-NOW.

A network of bacterial abundance was conducted at a species level. Bacteria that had correlated with and could promote the growth of each other are clearly shown in the network, respectively (R^2^ > 0.6, *P* ≤ 0.05, **Figure 7**).

### Random Forest Model Constructed to Distinguish PCOS and Control Women

**Figure 8:**
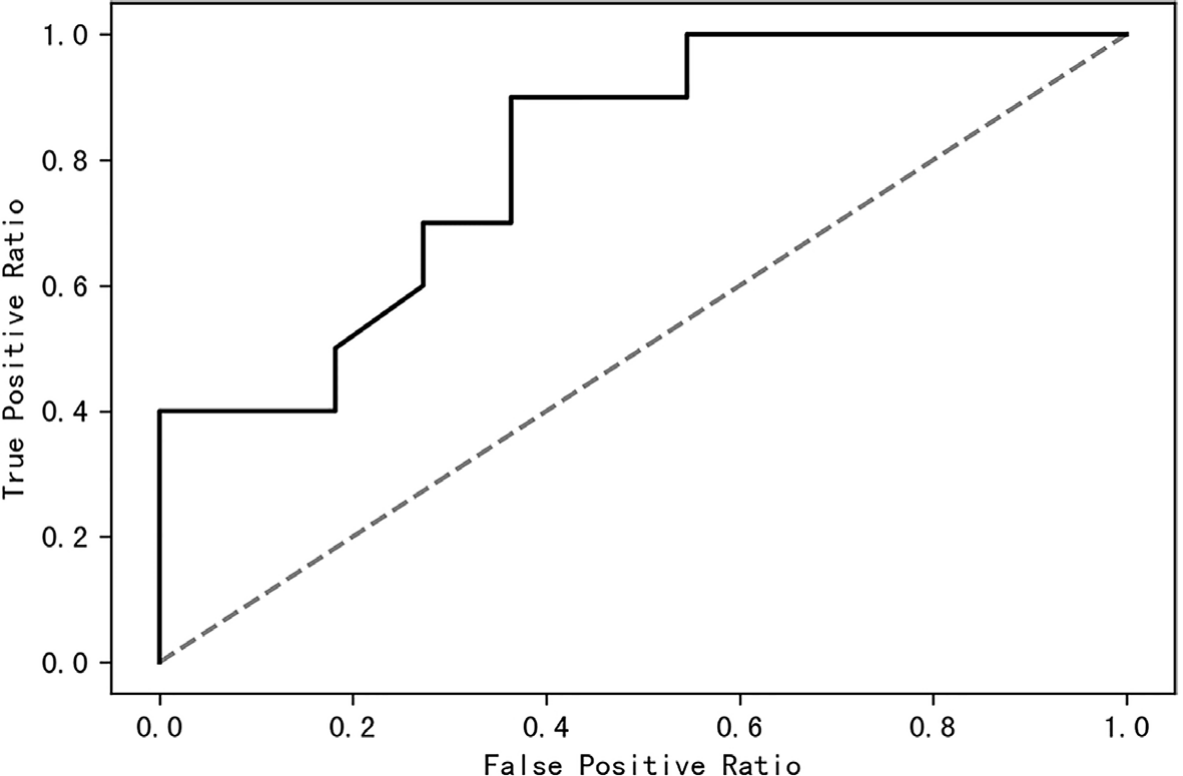
The top 10 species of contribution were used to construct a random forest model with an AUC of 0.87. AUC, area under the curve.

A receiver operating characteristic (ROC) curve was drawn to find a model that could distinguish women with PCOS from healthy control women. The area under the curve was 0.63 when a random forest model was constructed with all species; it increased to 0.87 when the top 10 contributing species were used (**Figure 8**). The top 10 contributing species are listed in **Table 2**.

**Table 2:** List of the top 10 species for contributions that were used in the random forest model.

Species	Contribution
Clostridiales_unclassified	0.138619
Fusicatenibacter saccharivorans	0.119748
Bacteroides vulgatus	0.112312
uncultured Lachnospiraceae bacterium	0.110208
Faecalibacterium prausnitzii	0.102373
Lachnospiraceae_unclassified	0.090574
Roseburia inulinivorans	0.089508
Alistipes putredinis	0.084156
Ruminococcaceae_unclassified	0.079673
Gemmiger formicilis	0.072829

## Discussion

We first studied the gut microbial community of PCOS patients by sequencing full-length 16S rDNA of the microbiota. In our study, participants were recruited and divided into PCOS and control groups; they were further divided into subgroups according to IR index, BMI and enterotype respectively. We analyzed clinical indexes. We noticed a trend that patients in PCOS-IR and PCOS-OW groups showed more disturbances in biochemical indexes, FINS, FPG, HOMA-IR, and TT when compared to PCOS-NIR and PCOS-NOW groups (**Supplementary Table 2**), though no significant statistical difference was observed. This indicated more glucose and lipid metabolism disorders may have been presented in PCOS-IR and PCOS-OW groups. LEfSe analysis of pathways (**Figure 6**) also showed fatty acid elongation and lipid biosynthesis were abundant in PCOS patients. In comparison, a trend of increased hypothalamic–pituitary–ovarian axis (HPOA) functional disorders in PCOS-NIR and PCOS-NOW groups was observed since they had higher LH and AMH levels than PCOS-IR and PCOS-OW groups. However, due to the restriction in sample numbers, more participants are needed for a more definitive conclusion. The reason for this observation may be a reduced negative feedback on the pituitary of E_2_ since we noticed that E_2_ levels of patients in PCOS-NIR and PCOS-NOW groups were lower than those of patients in control-NIR and control-NOW groups, and they showed higher level of LH than PCOS-IR and PCOS-OW, in which we hypothesized that their pituitary had a higher sensitivity to GNRH, or got less negative feedback on their pituitary due to low level of E_2_.

A significant difference between PCOS and control groups was not observed with regard to α diversity. Significant differences were observed when participants were sub-grouped by BMI and IR (**Figure 1**), indicating metabolic dysfunction added to the decrease in bacterium diversity. According to LEfSe analysis, *R. gnavus, Roseburia* sp. *11SE38, D. succinatiphilus, and L. bacterium 2_1_58FAA*, all from the *Firmicutes* phylum, were increased in patients with PCOS. The increased abundance of *Firmicutes* is associated with a high BMI (Husen et al., 2008); it was shown to be the most abundant phylum in obese women (Chávez-Carbajal et al., 2019), and was believed to participate in the occurrence of obesity. In our study, participants in PCOS group had a significantly higher BMI than non-PCOS women. *Lachnospiraceae* spp. and *Roseburia* spp. were found to be more abundant in women with obesity and metabolic syndrome (Chávez-Carbajal et al., 2019). *R. gnavus* utilizes glycans from the intestinal mucus layer as energy sources; it was significantly more abundant in patients with inflammatory bowel disease (IBD) when compared to a control group, and found to grow when IBD activity increased, which was explained as it had gene families involved in protection against the oxidative stress response in IBD gut (Hall et al., 2017). In the former study, women with PCOS, whether fat or lean, showed an increased reactive oxygen species level, which positively correlated with androgen secretion stimulated by human chorionic gonadotrophin (HCG) (González et al., 2019). The reason that *R. gnavus* was abundant in women with PCOS may be because its clade led to a tolerance of the oxidative stress environment found in the gut, as well as several genes that could help in the utilization of adhesion and mucus (Hall et al., 2017). *R. gnavus* was also observed to be associated with respiratory allergies and their increase before the onset of allergy symptoms (Gupta et al., 2015). Several *Roseburia* and *Lachnospiraceae* species are butyrate-producing colon bacteria, and play an important role in colonic health such as in improving the intestinal barrier. *Roseburia* sp. *11SE38* is from unclassified *Roseburia*, and *L. bacterium 2_1_58FAA* is from unclassified *Lachnospiraceae*, both exist mostly in patients with PCOS according to a heatmap (**Figure 4**), meaning it may have different effects from other “good” species in *Roseburia.* In a study by Raman et al. of non-alcoholic fatty liver disease, the amount of *Lachnospiraceae* and *Roseburia* was increased in such patients, which indicated that these bacteria may be associated with metabolic dysfunction in the body (Raman et al., 2013).

*P. stercorea* was in greater abundance in the PCOS group when compared to the control group (**Figures 3** and **4**). Moreover, it was increased in PCOS-NIR and PCOS-NOW groups when compared to control-NIR and control-NOW groups, respectively, suggesting *P. stercorea* may have a role in the pathological changes of PCOS in people with less metabolic disorder. Silvia et al. also showed that a hypocaloric diet led to an increase in the abundance of *P. stercorea* in obese and overweight patients (Pisanu et al., 2020). *P. copri* and *Prevotellaceae* spp. promoted the growth of each other in PCOS-NOW as well as PCOS-NIR groups (**Figure 7**). *P. copri* were more abundant in a rheumatoid arthritis than osteoarthritis group (Lee et al., 2019). In research by Dillon et al., the prevalence of mucosal *P. copri* and *P. stercorea* had a positive correlation with the expression of CD40 on colonic myeloid dendritic cells (mDCs). CD40 is positively associated with a mucosal HIV-1 viral load, and mucosal inflammatory cytokines, such as IL-23, IL-1β, IL-6, and tumor necrosis factor-α as well as with IL-10 levels. This indicates that *Prevotella* spp. may contribute to mucosal inflammation and the activation of immune disease (Dillon et al., 2016). The genus *Prevotella* shows an increased prevalence when more fiber is added to a diet (Kovatcheva-Datchary et al., 2015). People with *Prevotella*-dominated enterotype favored sugar, especially monosaccharides. Lipolytic and proteolytic fermentation were detected at lower levels in *Prevotella* enterotypes (Wu et al., 2011), indicating that diet may be the reason that such people weighed less than patients in PCOS-OW and PCOS-IR groups. John et al. (Lukens et al., 2014) showed that lean-fat diet (LFD) mice developed an outgrowth of *Prevotella* genera when compared to high-fat diet (HDF) mice; the osteomyelitis-related inflammatory factor, IL-1β, was increased in LDF mice. In the former study, the *Prevotellaceae* family was found to be decreased in patients with PCOS, especially in the PCOS-IR group, in a study by Zeng et al., (2019). In their study, people in the healthy control group had a higher relative abundance of *Prevotellaceae* than *Bacteroidaceae*, in contrast to the PCOS group. However, our data showed that the *Bacteroides* genus from the *Bacteroidaceae* family dominated in both control and PCOS groups. Twenty-six women in the control group and 31 women in the PCOS group had a *Bacteroides*-dominated enterotype, while only 11 women in the control group and 14 women in the PCOS group had a *Prevotella*-dominated enterotype. A reason for this difference may be differences in diets from northeast China (our research) and southwest China (Zeng’s research). Enterotypes are influenced by long-term dietary patterns. Eating protein-rich food, the bacteria in gut would tend to be dominated by Gram-positive bacteria while carbohydrate-rich food might lead to a Gram-negative-dominated gut environment (Wu et al., 2011). In our study, PCOS with *Prevotella*-dominated enterotype had a lower level of E_2_ than control with *Prevotella*-dominated enterotype, and they had a higher level of LH than PCOS with Bacteroides-dominated enterotype. Meanwhile we find both two enterotype of PCOS patients had higher level of BMI and FINS, HOMA-IR than control groups, and especially PCOS with Bacteroides-dominated enterotype showed significant changes of lipid metabolism status (**Table 1**). The enterotype may not intervene the mechanism that causes the pathogenesis, but it might be the external manifestation of different types of PCOS with different pathogenesis. We hypothesized that PCOS women with a *Bacteroides* enterotype in southwest China may lack fiber in their diet, thus increasing any dysfunction of lipid modulation and appearances of metabolic disorders. Women in northeast China showed an abnormally high level of *P. stercorea* from the *Prevotella* genus compared to healthy people; this may lead to or accelerate the pathological development of PCOS in patients, and, at the same time, retaining a normal lipid metabolism. *P. stercorea* is a Gram-negative bacterium. LEfSe difference analysis showed that CMP-3-deoxy-D-manno-octulosonate biosynthesis I, Kdo transfer to lipid IV_A_ III (*Chlamydia*), and super-pathway of GDP-mannose derived O antigen building block biosynthesis, which participate in the biosynthesis of a component of bacterial, lipopolysaccharide (LPS), were enriched in PCOS women (**Figure 6** and **Supplementary Figure 2**). Increased LPS production from Gram-negative gut bacteria contributes to metabolic aberrations when mucosal barriers weaken. The gut leak makes it convenient for LPS to enter into the circulation, which promotes the development of metabolic endotoxemia, and accelerates obesity, insulin resistance, and other metabolic disturbances (d’Hennezel et al., 2017). More study will be needed in the future regarding the role of *P. stercorea* in the pathogenesis of lean PCOS, and the relationship of diet in different areas of China with enterotype. This may allow the correction of inflammatory disorders in PCOS patients by adjusting their diets.

Our data appeared to be similar to those of articles reported by Liu (Liu et al., 2017), Torres (Torres et al., 2018), Zeng (Zeng et al., 2019), Zhang (Zhang et al., 2019), Qi (Qi et al., 2019), and Chu (Chu et al., 2020) in that bacteria from the *Bacteroides* family showed an increased abundance in patients with PCOS. Here we identified several specific species from *Bacteroides* that showed a difference in abundance between groups. *B. fragilis* was significantly increased in PCOS patients with an LDA of score more than 3. *B. fragilis* is a common anaerobe in extraintestinal infections (Valguarnera and Wardenburg, 2020), and enterotoxigenic *B.fragilis* may contribute to systemic inflammation (Sun et al., 2019). *Bacteroides xylanisolvens* showed a greater abundance in PCOS-IR when compared to control-IR. It was found to be specialized in the degradation of xylans with low complexity (Despres et al., 2016). *Christensenellaceae* spp. were shown to have increased relative abundance in the control group. The relative abundance of the *Christensenellaceae* family has a negative relationship with BMI, LDL, and apolipoprotein B as well as features of metabolic syndrome such as obesity and hypertriglyceridemia (Waters and Ley, 2019; Li et al., 2020), and was positively associated with HDL (Fu et al., 2015; Hibberd et al., 2019). It is reported to be higher in people who have a balanced omnivorous diet than in vegetarians (De Filippis et al., 2016).

Our method of library construction is suitable for the detection of bacterial colony structure of all types of samples. The 16S of bacteria has a total length of about 1500bp and contains 9 variable regions. In the past, 16S sequencing only selected one or two variable regions, and the length was only 3/400bp. The detection of full length of all of the 9 variable regions upgraded the identification of flora structure to “species” level from the traditional way which usually resulted in “genus” level, determining the ecological structure of environmental microorganisms more accurately, which is convenient for in-depth research. Therefore, the samples can be tested for specific bacterial species. Moreover, compared with Pacbio and other third-generation sequencing technologies, the reads sequence obtained by Illumina sequencing technology is of higher quality and can obtain more accurate DNA sequence. Based on high-throughput sequencing technology, a large number of samples can be analyzed at once by adding different tag sequences to each sample. In this way, the abundance of the detected microbiota could have a good fidelity.

## Conclusion

Our research used 16S-FAST technology for the first time to study the characteristics and differences in the gut microbiota of patients with PCOS and healthy controls. Several bacterial species with different abundances as well as differences in metabolic pathways were detected in the PCOS group. We found that levels of *R. gnavus, Roseburia* spp., and *Lachnospiraceae* spp. were higher in PCOS patients. We also found that the level of *P. stercorea* was significantly higher in PCOS-NIR and PCOS-NOW groups compared to control-NIR and control-NOW groups, and may be involved in the pathogenesis of lean PCOS patients in northeast China. PCOS with *Prevotella* enterotype showed similar clinical indexes level with PCOS-NIR and PCOS-NOW groups, and PCOS with *Bacteroides* enterotype showed similar clinical indexes level with PCOS-IR and PCOS-OW groups. The intestinal flora may be regarded a new treatment site that can be regulated by diet and drugs to interfere with the occurrence and development of, or to improve, PCOS.

## Data Availability Statement

The datasets presented in this study can be found in online repositories. The names of the repository/repositories and accession number(s) can be found below: NCBI SRA, PRJNA694729.

## Ethics Statement

This study protocol was approved by the Ethics Committee of Shengjing Hospital affiliated to China Medical University. The patients/participants provided their written informed consent to participate in this study. Written informed consent was obtained from the individual(s) for the publication of any potentially identifiable images or data included in this article.

## Author Contributions

JJ and XW contributed to the conception of the study. KY performed the sequencing. ZW contributed significantly to data analyses. SD performed the data analyses and wrote the manuscript. SJ, GL, WZ, CL, and DL helped perform the analysis with constructive discussions. All authors contributed to the article and approved the submitted version.

## Funding

This work was supported by the National Natural Science Foundation of China (No.81671423 and No.81402130).

## Conflict of Interest

The authors declare that the research was conducted in the absence of any commercial or financial relationships that could be construed as a potential conflict of interest.
