# Supplementary figures and images for: 16S rDNA Full-Length Assembly Sequencing Technology Analysis of Intestinal Microbiome in Polycystic Ovary Syndrome
Source: Front Cell Infect Microbiol. 2021 May 10;11:634981. doi: 10.3389/fcimb.2021.634981 (PMC8141595; doi:10.3389/fcimb.2021.634981)

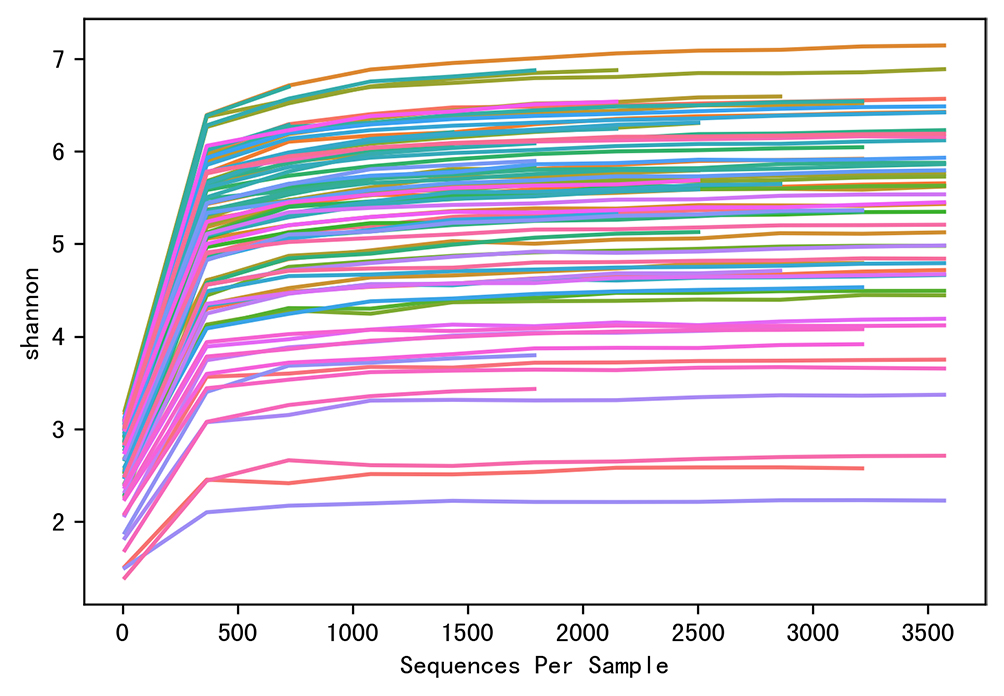

Supplement: Supplementary file 1 [file Image_1.tif]

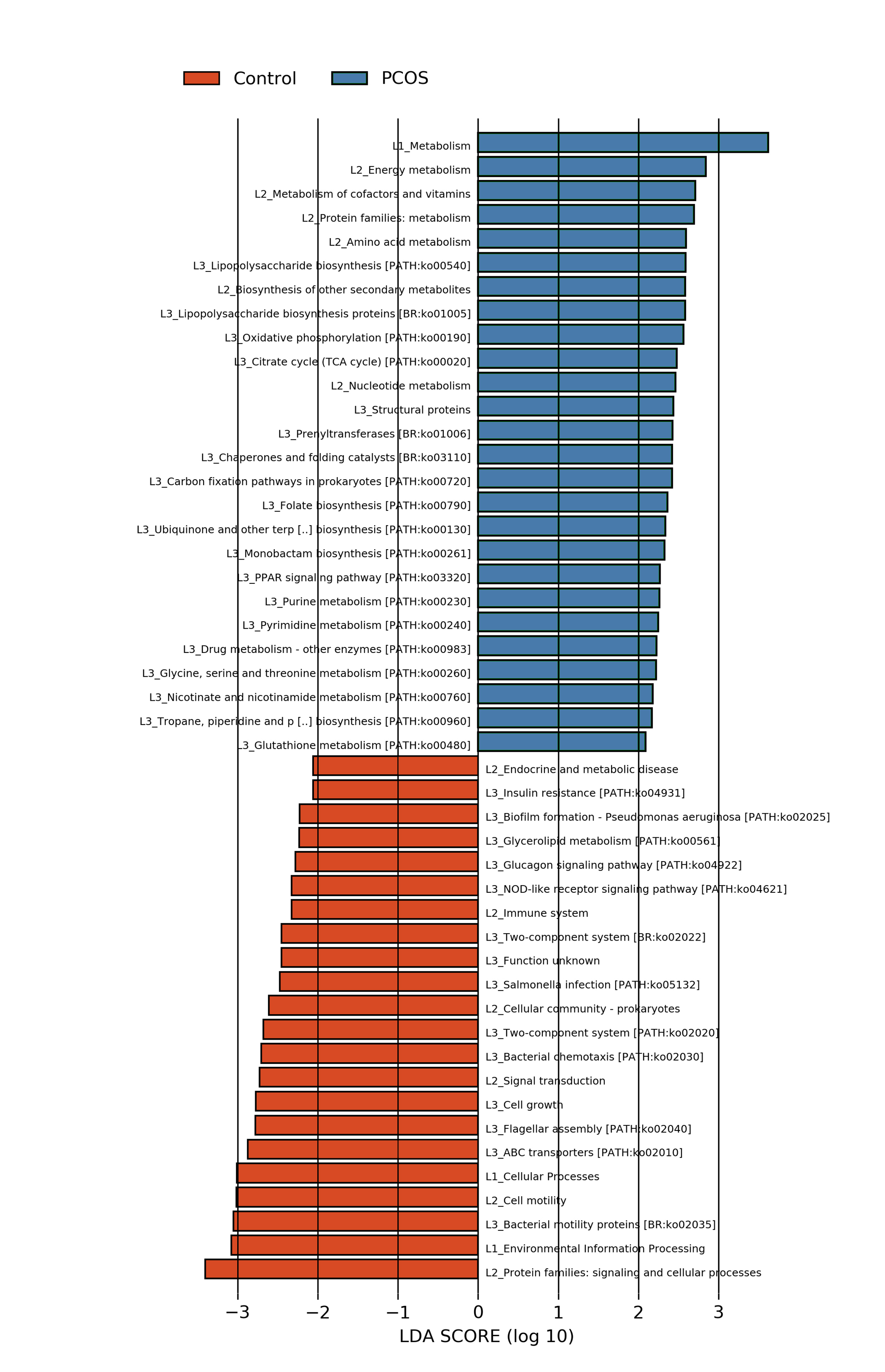

Supplement: Supplementary file 2 [file Image_2.tif]
