# Supplementary material for: 16S rDNA Full-Length Assembly Sequencing Technology Analysis of Intestinal Microbiome in Polycystic Ovary Syndrome
Source: Front Cell Infect Microbiol. 2021 May 10;11:634981. doi: 10.3389/fcimb.2021.634981 (PMC8141595; doi:10.3389/fcimb.2021.634981)
Supplement: Supplementary file 3 [file Table_1.docx]

Supplementary Table 1. List of changed communities of microbiota in PCOS patients

|  | Year | Article | Area | Sequencing  method | Participants | up regulated in PCOS | down regulated in PCOS |
| --- | --- | --- | --- | --- | --- | --- | --- |
| 1 | 2017 | (Lindheim et al., 2017) | Graz, Austria | V1-V2 regions of 16S rDNA amplicons sequencing | control (n=19)  PCOS (n=24) |  | Order：  ML615J-28  Family：  S24-7 |
| 2 | 2017 | (Liu et al., 2017) | Shanghai, China | V3-V4 regions of 16S rDNA amplicons sequencing | control (n=15)（obesity n=6，non-obesity n=9）  PCOS (n=33）（obesity n=21，non-obesity n=12） | Genus:  *Bacteroides*  *Escherichia/Shigella*  *Streptococcus*  *Blautia*  *Parabacteroides*  *Clostridium XIVa*  *Alistipes*  *Weissella*  *Granulicatella*  *Peptostreptococcus*  *Rothia* | Genus：  *Akkermansia*  *Ruminococcus*  *Alistipes*  *Coprococcus* |
| 3 | 2018 | (Torres et al., 2018) | California, America | V4 region of 16S rDNA amplicons sequencing | control (n=48)  PCOM (n=42)  PCOS (n=73) | Genus:  *Porphyromonas*  *Blautia*  Species:  *Bacteroides coprophilus*  *Faecalibacterium prausnitzii* | Genus：  *Anaerococcus*  *Odoribacter*  *Roseburia*  Specie：  *Ruminococcus bromii* |
| 4 | 2018 | (Insenser et al., 2018) | Rome, Italy | V4 region of 16S rDNA amplicons sequencing | nonhyperandrogenic control women(n=16)  PCOS (n=16)  control men (n=15) | Genus:  *Catenibacterium*  *Kandleria* |  |
| 5 | 2019 | (Zeng et al., 2019) | Zigong, China | V3-V4 regions of 16S rDNA amplicons sequencing | control （n=8）  NIR-PCOS (n=8)  IR-PCOS (n=9) | Species:  *Bacteroides plebeius*  *Bacteroides uniformis*  *Bacteroides ovatus*  *Ruminococcus 2*  Ruminococcus bicirculans | Genus：  *Prevotella 9* |
| 6 | 2019 | (Zhang et al., 2019) | Zigong, China | whole metagenome shotgun sequencing | control (n=26)  PCOS (n=38) | Genus：  *Clostridium*  *Parabacteroides*  *Lactobacillus*  *Bacteroides*  *Oscillibacter*  *Escherichia/Shigella*  Metagenomic species：  *Subdoligranulum variabile MGS031*  *Prevotella copri MGS035*  *Eubacterium rectale MGS044*  *Eubacterium eligens MGS013*  *Dialister invisus MGS048*  *Collinsella aerofaciens MGS060*  *Bacteroides vulgatus MGS014*  *Bacteroides uniformis MGS022*  *Bacteroides sp. MGS027*  *Bacteroides caccae MGS018*  *Alistipes putredinis MGS041* | Genus：  *Faecalibacterium*  *Lachnospira*  *Bifidobacterium*  *Blautia*  Metagenomic species：  *Bifidobacterium sp. MGS003*  *Faecalibacterium prausnitzii MGS004*  *Bacteroides sp. MGS008*  *Bifidobacterium animalis MGS015*  *Roseburia inulinivorans MGS148*  *Coprococcus eutactus MGS007*  *Bifidobacterium pseudocatenulatum MGS052*  *Roseburia intestinalis MGS026*  *Bacteroides stercoris MGS016*  *Coprococcus comes MGS023*  *Bacteroides plebeius MGS006*  *Faecalibacterium prausnitzii MGS032* |
| 7 | 2019 | (Qi et al., 2019) | Beijing, China | whole metagenome shotgun sequencing | control (n=43)  PCOS (n=50) | Specie：  *Bacteroides vulgatus* |  |
| 8 | 2020 | (Zhou et al., 2020) | Shanghai, China | V3-V4 regions of 16S rDNA amplicons sequencing | control non-obese (n=30)  control obese (n=11)  PCOS non-obese (n=30)  PCOS obese (n=30) | Genus：  *Prevotella_7*  *Coprococcus_2*  *Lactobacillus*  *Lactococcus*  *Paraprevotella*  *Alloprevotella* | Genus：  *Synergistets*  *Coprococcus*  *Paraprevotella*  *Lactococcus*  *Alloprevotella*  *Holdmanella*  *Harryflintia*  *Lactobacillus*  *Prevotella*  *Clostridiales*  *Lachnospiraceae*  *Mollicutes*  *Neisseria*  *Hydrogenoanaerabcterium*  *Ruminococcaceae*  *Sellimonas* |
| 9 | 2020 | (Chu et al., 2020) | Shanghai, China | whole metagenome shotgun sequencing | control (n=14)  PCOS (n=14) | Species：  *Bacteroides sp_D20*  *Bacteroides fragilis_CAG_558*  *Escherichia coli*  *Escherichia sp_1_1_43*  *Escherichia sp_4_1_40B*  *Shigella sonnei*  *Shigella boydii*  *Shigella flexneri*  *Shigella sp_PAMC_28760*  *Shigella dysenteriae*  *Shigella sp_SF_2015*  *Enterobacteria phage SfV*  *Parabacteroides merdae*  *Ruminococcus sp_CAG_353*  *Comamonas kerstersii*  Metagenomic species:  *Parabacteroides merdae*  *Clostridiales*  *Enterobacteriaceae*  *Bacteroides fragilis*  *Bacteroides*  *Bilophila* | Species：  *Blautia hydrogenotrophica*  *Bacteroides sp_CAG_20*  *Bacteroides gallinarum*  *Bacteroides massiliensis*  *Tannerella sp_6_1_58FAA_CT1*  *Klebsiella pneumoniae*  *Barnesiella intestinihominis*  *Faecalibacterium prausnitzii*  *Alistipes obesi*  Metagenomic species:  *Faecalibacterium prausnitzii L2–6*  *Parabacteroides goldsteinii CL02T12C30*  *Parabacteroides goldsteinii CL02T12C30*  *Bacteroidales*  *Alistipes indistinctus YIT 12060*  *Bacteroides intestinalis DSM 17393* |
| 10 | 2020 | (Jobira et al., 2020) | Colorado, America | V3-V4 regions of 16S rDNA amplicons sequencing | control (n=21)  PCOS (n=37) | Families:  *Streptococcaceae* | Families:  *Bacteroidaceae*  *Porphyromonadaceae* |
| 11 | 2020 | (Liang et al., 2020) | Nanjing, China | V3-V4 regions of 16S rDNA amplicons sequencing | control non-obese (n=9)  PCOS non-obese (n=10)  PCOS obese (n=8) | Genus:  *Megamonas*  *Subdoligranulum* | Genus:  *Lachnospiraceae_UCG-010* |
| 12 | 2020 | (Haudum et al., 2020) | Graz, Austria | V1-V2 regions of 16S rDNA amplicons sequencing | control (n=25)  PCOS (n=25) | Genus:  *Bacteroides_186841*  *Megasphaera_590251*  *Megasphaera_357302*  *Ruminococcus_369338*  Strain:  *Torques_590251* | Families:  *S24-7_844589*  *Ruminococcaceae_368692*  Genus:  *Prevotella_172962*  *Prevotella_546557*  *Coprococcus_592980* |
| 13 | 2020 | (Eyupoglu et al., 2020) | Ankara, Turkey | V3-V4 regions of 16S rDNA amplicons sequencing | control obese (n=15)  PCOS obese (n=17) | Family:  *Ruminococcaceae* |  |
| 14 | 2021 | (Garcia-Beltran et al., 2021) | Barcelona, Spain | V3-V4 regions of 16S rDNA amplicons sequencing | control non-obese (n=31)  PCOS non-obese (n=30) | Family:  *XI* | Family:  *Prevotellaceae*  Genus  *Prevotella*  *Senegalimassilia* |
| 15 | 2021 | (Mammadova et al., 2021) | Ankara, Turkey | V3-V4 regions of 16S rDNA amplicons sequencing | control non-obese (n=22)  PCOS non-obese (n=24) | Families:  *Erysipelotrichaceae*  *Enterobacteriaceae*  *Planococcaceae*  *Gemellaceae* | Families:  *Peptostreptococcaceae* |
| 16 | 2021 | (Liang et al., 2021) | Guangzhou, China | V4 regions of 16S rDNA amplicons sequencing | control non-obese (n=10)  control obese (n=10)  PCOS non-obese (n=10)  PCOS obese (n=10) | Species:  *Parabacteroides distasonis*  *Escherichia coli*  *Bacteroides fragilis*  *Parabacteroides distasonis* |  |

Chu, W., Han, Q., Xu, J., Wang, J., Sun, Y., Li, W., et al. (2020). Metagenomic analysis identified microbiome alterations and pathological association between intestinal microbiota and polycystic ovary syndrome. *Fertil Steril* 113(6)**,** 1286-1298 e1284. doi: 10.1016/j.fertnstert.2020.01.027.

Eyupoglu, N.D., Ergunay, K., Acikgoz, A., Akyon, Y., Yilmaz, E., and Yildiz, B.O. (2020). Gut Microbiota and Oral Contraceptive Use in Overweight and Obese Patients with Polycystic Ovary Syndrome. *J Clin Endocrinol Metab* 105(12). doi: 10.1210/clinem/dgaa600.

Garcia-Beltran, C., Malpique, R., Carbonetto, B., González-Torres, P., Henares, D., Brotons, P., et al. (2021). Gut microbiota in adolescent girls with polycystic ovary syndrome: Effects of randomized treatments. *Pediatr Obes* 16(4)**,** e12734. doi: 10.1111/ijpo.12734.

Haudum, C., Lindheim, L., Ascani, A., Trummer, C., Horvath, A., Münzker, J., et al. (2020). Impact of Short-Term Isoflavone Intervention in Polycystic Ovary Syndrome (PCOS) Patients on Microbiota Composition and Metagenomics. *Nutrients* 12(6). doi: 10.3390/nu12061622.

Insenser, M., Murri, M., Del Campo, R., Martinez-Garcia, M.A., Fernandez-Duran, E., and Escobar-Morreale, H.F. (2018). Gut Microbiota and the Polycystic Ovary Syndrome: Influence of Sex, Sex Hormones, and Obesity. *J Clin Endocrinol Metab* 103(7)**,** 2552-2562. doi: 10.1210/jc.2017-02799.

Jobira, B., Frank, D.N., Pyle, L., Silveira, L.J., Kelsey, M.M., Garcia-Reyes, Y., et al. (2020). Obese Adolescents With PCOS Have Altered Biodiversity and Relative Abundance in Gastrointestinal Microbiota. *J Clin Endocrinol Metab* 105(6)**,** e2134-2144. doi: 10.1210/clinem/dgz263.

Liang, Y., Ming, Q., Liang, J., Zhang, Y., Zhang, H., and Shen, T. (2020). Gut microbiota dysbiosis in polycystic ovary syndrome: association with obesity - a preliminary report. *Can J Physiol Pharmacol* 98(11)**,** 803-809. doi: 10.1139/cjpp-2019-0413.

Liang, Z., Di, N., Li, L., and Yang, D. (2021). Gut microbiota alterations reveal potential gut-brain axis changes in polycystic ovary syndrome. *J Endocrinol Invest*. doi: 10.1007/s40618-020-01481-5.

Lindheim, L., Bashir, M., Munzker, J., Trummer, C., Zachhuber, V., Leber, B., et al. (2017). Alterations in Gut Microbiome Composition and Barrier Function Are Associated with Reproductive and Metabolic Defects in Women with Polycystic Ovary Syndrome (PCOS): A Pilot Study. *PLoS One* 12(1)**,** e0168390. doi: 10.1371/journal.pone.0168390.

Liu, R., Zhang, C., Shi, Y., Zhang, F., Li, L., Wang, X., et al. (2017). Dysbiosis of Gut Microbiota Associated with Clinical Parameters in Polycystic Ovary Syndrome. *Front Microbiol* 8**,** 324. doi: 10.3389/fmicb.2017.00324.

Mammadova, G., Ozkul, C., Yilmaz Isikhan, S., Acikgoz, A., and Yildiz, B.O. (2021). Characterization of gut microbiota in polycystic ovary syndrome: Findings from a lean population. *Eur J Clin Invest* 51(4)**,** e13417. doi: 10.1111/eci.13417.

Qi, X., Yun, C., Sun, L., Xia, J., Wu, Q., Wang, Y., et al. (2019). Gut microbiota-bile acid-interleukin-22 axis orchestrates polycystic ovary syndrome. *Nat Med* 25(8)**,** 1225-1233. doi: 10.1038/s41591-019-0509-0.

Torres, P.J., Siakowska, M., Banaszewska, B., Pawelczyk, L., Duleba, A.J., Kelley, S.T., et al. (2018). Gut Microbial Diversity in Women With Polycystic Ovary Syndrome Correlates With Hyperandrogenism. *J Clin Endocrinol Metab* 103(4)**,** 1502-1511. doi: 10.1210/jc.2017-02153.

Zeng, B., Lai, Z., Sun, L., Zhang, Z., Yang, J., Li, Z., et al. (2019). Structural and functional profiles of the gut microbial community in polycystic ovary syndrome with insulin resistance (IR-PCOS): a pilot study. *Res Microbiol* 170(1)**,** 43-52. doi: 10.1016/j.resmic.2018.09.002.

Zhang, J., Sun, Z., Jiang, S., Bai, X., Ma, C., Peng, Q., et al. (2019). Probiotic Bifidobacterium lactis V9 Regulates the Secretion of Sex Hormones in Polycystic Ovary Syndrome Patients through the Gut-Brain Axis. *mSystems* 4(2). doi: 10.1128/mSystems.00017-19.

Zhou, L., Ni, Z., Cheng, W., Yu, J., Sun, S., Zhai, D., et al. (2020). Characteristic gut microbiota and predicted metabolic functions in women with PCOS. *Endocr Connect* 9(1)**,** 63-73. doi: 10.1530/ec-19-0522.
