# Supplementary material for: 16S rDNA Full-Length Assembly Sequencing Technology Analysis of Intestinal Microbiome in Polycystic Ovary Syndrome
Source: Front Cell Infect Microbiol. 2021 May 10;11:634981. doi: 10.3389/fcimb.2021.634981 (PMC8141595; doi:10.3389/fcimb.2021.634981)
Supplement: Supplementary file 4 [file Table_2.docx]

Supplementary Table 2. Clinical parameters of participants in the study

|  | control | PCOS | P | control | | PCOS | | control | | PCOS | |
| --- | --- | --- | --- | --- | --- | --- | --- | --- | --- | --- | --- |
|  |  |  |  | NIR(n=22) | IR(n=7) | NIR(n=15) | IR(n=27) | NOW(n=25) | OW(n=12) | NOW(n=14) | OW(n=31) |
| Age(year) | 31(28-33.5) | 30(27-34) | 0.22 | 32(29.5-34) | 31(28-32) | 28(26-36) | 30(26.75-32.25) | 31(28-35) | 31(28.5-32.25) | 30.5(25.75-35) | 30(27-33) |
| Height(m) | 162(160-165.5) | 162(160-166) | 0.86 | 162(159.25-165) | 165(158.5-168) | 163(158-167) | 162.5(160-165.25) | 162(160-165) | 162.5(159-168.5) | 162.5(158-166.25) | 162(160-165.5) |
| Weight(m) | 60(54.5-67.5) | 72.5(60-82.5) | <0.001 | 59.5(55-65) | 70(56.8-75) | 58(50-67.5) | 80(69.5-87.88) | 55(52-60) | 69(65.9-75.5) | 56.5(50-60) | 78.5(69.5-85.75) |
| BMI(kg/m2) | 22.58(20.14-25.09) | 26.95(22.89-31.25) | <0.001 | 22.45(20.91-24.1) | 25.4(21.02-27.81) | 22.1(19.38-25.1) | 29.29(26.23-31.95) | 21.28(19.81-22.58) | 25.63(24.83-26.88) | 21.83(19.3-22.85) | 29.03(26.17-31.87) |
| Waistline(cm) | 77(73-83) | 88.5(79.5-99) | <0.001 | 75.5(72.25-83) | 80(75.5-87) | 78(73-88) | 94(86.5-102.75)^b^ | 76(73-81) | 82.5(75.5-91.5) | 76(71.5-80.5) | 94(88-101.5)^d^ |
| Hipline(cm) | 95(91-100) | 100(94-107.5) | 0.013 | 94.5(91-98.75) | 101(97-103.5) | 93(89-100) | 105(96.75-112.25) | 93(90-95) | 101(99-105.5) | 90(89-94.5) | 105(98-112.5) |
| WHR | 0.81(0.78-0.86) | 0.87(0.84-0.94) | <0.001 | 0.82(0.78-0.86) | 0.81(0.79-0.9) | 0.84(0.82-0.87) | 0.91(0.86-0.94)^b^ | 0.81(0.78-0.86) | 0.81(0.78-0.89) | 0.84(0.79-0.86) | 0.9(0.86-0.94)^d^ |
| TP(g/L) | 74(70.45-76.05) | 76.75(74.48-79.88) | <0.001 | 74.15(70.53-76.15) | 72.8(69.2-76.3) | 73.9(71.98-76.33) | 78.2(76.58-80.55)^b^ | 73.5(70-76.8) | 74.6(71-75.7) | 76.85(73.85-78.9) | 76.75(74.88-80.25) |
| ALB(g/L) | 45.7(43.15-48.05) | 47.35(45.45-50.1) | 0.019 | 45.3(  42.8-48) | 46.4(44.7-48.9) | 46.3(44.6-51.05) | 47.5(45.68-50.1) | 45.6(42.45-48.05) | 46.4(44.4-48.23) | 48.1(44.35-51.35) | 47.1(45.63-49.45) |
| A/G | 1.65(1.54-1.77) | 1.62(1.46-1.73) | 0.578 | 1.62(1.47-1.72) | 1.82(1.6-1.98) | 1.69(1.62-1.93) | 1.57(1.45-1.72) | 1.61(1.49-1.72) | 1.71(1.58-1.87) | 1.69(1.47-1.91) | 1.58(1.46-1.72) |
| AST(U/L) | 16(13-17) | 19.3(16-24.5) | <0.001 | 16.5(14-18.5) | 13(11-15.5) | 18.5(14.75-23.13) | 20(16-27.5)^b^ | 16(12.5-18) | 14.6(13-16.75) | 18.8(16-21.88) | 19.5(16-26)^d^ |
| ALT(U/L) | 13(10.3-15) | 21(14.15-39.5) | <0.001 | 13(10-15.75) | 12(11-14) | 13.8(9.5-20.25) | 29(17-43)^b^ | 13(9.5-15) | 13(11.25-15.5) | 15.8(9-24.25) | 28.5(16.25-45)^d^ |
| GGT(U/L) | 12(11-18.1) | 24.5(16.5-37.5) | <0.001 | 12(10.25-17.5) | 14(11-21) | 17(12.75-21.5) | 30(19.75-42.25)^b^ | 12(10-15) | 16.6(11.5-21.5) | 15.5(12-18.53) | 28(20-41.75)^d^ |
| ALP(U/L) | 65.5(57.85-71.25) | 78.5(65.58-94.2) | <0.001 | 64.3(55.23-68.95) | 70.9(60.7-75.55) | 66.35(55.83-79.28) | 86.9(71.63-97.78) | 62.2(57.45-68.8) | 68.7(59.73-73.45) | 67.2(58-77.03) | 86.9(69.65-97.08) |
| PALB(g/L) | 0.25(0.23-0.27) | 0.28(0.24-0.31) | 0.025 | 0.25(0.22-0.27) | 0.27(0.26-0.29) | 0.24(0.21-0.26) | 0.29(0.26-0.33) | 0.25(0.22-0.27) | 0.26(0.24-0.29) | 0.25(0.22-0.34) | 0.28(0.26-0.31) |
| CHE(U/L) | 7588(6559-8820) | 9131(8286-10528) | <0.001 | 7457(6545-8811) | 7861(6711.5-9382) | 8214(7055.5-8794.5) | 9991.5(8671.5-11217.25)^b^ | 7178(6534-8393.5) | 8621(7861-8986) | 8311(7435-8837) | 9762(8635.5-11152.25) |
| TBIL(umol/L) | 11.2(10.15-15.8) | 10(8.6-12.93) | 0.056 | 11.15(9.38-16.15) | 11.3(10.45-16.1) | 10.3(9.4-15.45) | 9.85(8.4-12.33) | 11.1(9.45-17.6) | 11.25(10.63-13.98) | 12.1(9.7-19.28) | 9.8(8.45-11.95) |
| DBIL(umol/L) | 3.4(2.55-4.5) | 2.7(1.95-3.3) | 0.006 | 3.2(2.3-4.5) | 3.7(3-4.7) | 2.55(2-3.93) | 2.7(1.9-3.3)^b^ | 3.1(2.4-4.65) | 3.4(3.2-3.9) | 2.65(1.63-4.18) | 2.7(2.1-3.25) |
| IDBIL(umol/L) | 8.4(7.25-11.3) | 8(6.05-9.5) | 0.175 | 8.7(7.1-13.4) | 7.6(7.4-11.1) | 8.2(7.05-11.4) | 7.55(5.98-9.5) | 8.5(6.85-13.6) | 7.6(7.4-10.7) | 9.1(7.48-15.75) | 7.4(5.93-8.95) |
| TBA(umol/L) | 2.08(1.39-3.3) | 2.34(1.55-3.73) | 0.577 | 2.52(1.44-3.41) | 1.8(1.31-2.74) | 2.35(1.24-4.79) | 2.31(1.62-3.16) | 2.52(1.66-3.79) | 1.77(1.28-2.13) | 2.4(1.07-6.45) | 2.34(1.78-3.06) |
| MAO(U/L) | 5.22(4.5-6.4) | 6.58(5.59-8.23) | 0.019 | 5.13(4.09-6.33) | 5.94(5.17-7.13) | 4.5(4.03-5.96) | 6.96(6.33-8.54) | 5.13(4.17-6.38) | 5.91(4.97-6.81) | 5.41(4.13-6.09) | 6.78(6.03-8.41) |
| CHOL(mmol/L) | 4.32(4.12-4.92) | 4.67(4.34-5.78) | 0.031 | 4.34(4.13-5.07) | 4.28(3.96-4.48) | 4.56(4.08-4.93) | 4.77(4.42-5.8) | 4.29(4.11-4.67) | 4.37(4.19-5.18) | 4.87(4.4-6.23) | 4.64(4.21-5.78) |
| TG(mmol/L) | 0.81(0.59-1.16) | 1.39(0.87-2.27) | 0.001 | 0.77(0.54-1.09) | 0.87(0.67-1.32) | 0.82(0.48-1.14) | 1.99(1.15-2.46)^b^ | 0.71(0.55-0.88) | 1.16(0.63-1.73) | 0.83(0.5-1.18) | 1.52(1.04-2.32) |
| HDL-C(mmol/L) | 1.45(1.15-1.84) | 1.13(0.98-1.4) | 0.014 | 1.5(1.16-1.85) | 1.19(1.06-1.76) | 1.44(1.27-2) | 1.07(0.95-1.25) | 1.58(1.16-1.89) | 1.2(0.97-1.57) | 1.37(1.17-2.21) | 1.06(0.95-1.32) |
| LDL-C(mmol/L) | 2.58(2.28-3.25) | 2.94(2.36-3.74) | 0.094 | 2.6(2.28-3.27) | 2.41(2.03-3.03) | 2.72(2.14-3.25) | 3.03(2.46-4.03) | 2.4(2.23-3.16) | 2.8(2.44-3.38) | 2.76(2.19-4) | 2.96(2.36-3.69) |
| apoA1(g/L) | 1.52(1.33-1.68) | 1.39(1.24-1.55) | 0.107 | 1.56(1.38-1.69) | 1.41(1.18-1.8) | 1.54(1.36-1.6) | 1.34(1.22-1.52) | 1.54(1.41-1.7) | 1.41(1.14-1.68) | 1.56(1.37-1.69) | 1.35(1.22-1.53) |
| apoB(g/L) | 0.71(0.62-0.87) | 0.92(0.79-1.14) | <0.001 | 0.69(0.62-0.92) | 0.75(0.61-0.85) | 0.82(0.74-1.07) | 1.01(0.82-1.16)^b^ | 0.66(0.6-0.85) | 0.81(0.65-0.92) | 0.96(0.81-1.12)^c^ | 0.92(0.77-1.15) |
| sd-LDL(mmol/L) | 0.66(0.53-1.1) | 0.98(0.83-1.47) | 0.001 | 0.71(0.52-1.12) | 0.64(0.57-0.9) | 0.79(0.52-0.91) | 1.1(0.94-1.53)^b^ | 0.57(0.51-0.82) | 0.77(0.62-1.19) | 0.84(0.61-1.54) | 1.01(0.88-1.44) |
| FPG(mmol/L) | 5.24(4.85-5.45) | 5.21(4.9-5.65) | 0.66 | 5.22(4.85-5.48) | 5.33(4.96-5.39) | 4.92(4.55-5.21) | 5.39(5-5.91) | 5.24(4.84-5.42) | 5.29(5.03-5.52) | 4.99(4.55-5.32) | 5.39(4.94-5.88) |
| 25(OH)-VitD(ng/mL) | 12.53(10.28-17.29) | 12.95(9.75-16.49) | 0.737 | 13.67(10.13-18.33) | 12.04(9.88-15.01) | 14.43(9.62-20.59) | 11.42(9.82-15.45) | 12.32(9.5-15.86) | 15.9(11.92-20.11) | 14.43(10.82-18.68) | 11.4(9.59-15.54) |
| Prog(ng/mL) | 0.59(0.34-0.74) | 0.45(0.23-0.64) | 0.088 | 0.58(0.32-0.71) | 0.77(0.33-0.98) | 0.45(0.26-0.55) | 0.44(0.21-0.69) | 0.6(0.42-0.74) | 0.41(0.23-0.89) | 0.49(0.34-0.62) | 0.41(0.21-0.68) |
| PRL(ng/mL) | 12.28(9.75-15.37) | 9.22(6.4-13.64) | 0.024 | 12.43(9.92-15.37) | 12.28(9.06-17.22) | 11.85(7.64-15.34) | 7.94(5.81-13) | 13.04(10.03-15.84) | 10.26(9.29-13.66) | 12.3(10.07-16.24) | 7.72(5.97-12.24) |
| SHBG(nmol/L) | 55.9(35.55-75.7) | 17.4(12.3-33.8) | <0.001 | 63.85(43.88-92.65) | 36.2(25.8-51.45) | 41.3(30.3-51.6) | 15.2(11.85-20)^b^ | 72.4(58.8-94.7) | 35.55(26.13-42.78) | 41.3(33.75-49.7) | 15.2(11.65-20.55)^d^ |
| FAI(%) | 2.88(1.93-4.52) | 13.39(6.81-19.4) | <0.001 | 2.48(1.81-3.94) | 4.52(2.45-5.5) | 4.72(3.87-7.9) | 17.39(9.43-26.05)^b^ | 2.01(1.52-3.07) | 4.52(3.21-6.12) | 4.72(4.11-7.85) | 17.03(8.04-24.48)^d^ |
| TT(ng/mL) | 0.42(0.37-0.56) | 0.69(0.51-0.82) | <0.001 | 0.43(0.37-0.59) | 0.42(0.35-0.51) | 0.6(0.41-0.72) | 0.71(0.53-0.84)^b^ | 0.42(0.35-0.54) | 0.43(0.38-0.68) | 0.63(0.5-0.77) | 0.7(0.51-0.84) |
| LH(mIU/mL) | 3.82(2.9-4.89) | 8.37(4.75-10.88) | <0.001 | 4.04(2.96-5.1) | 3.27(2.39-4.1) | 9.51(4.34-12.2)^a^ | 8.01(5.2-9.76)^b^ | 4.28(2.98-5.3) | 3.54(2.61-3.95) | 9.51(6.46-12.53)^c^ | 8.24(4.34-9.78)^d^ |
| FSH(mIU/mL) | 7.08(6.43-8.38) | 6.22(5.4-7.33) | 0.01 | 6.9(6.02-8.4) | 7.16(6.99-8.33) | 6.67(5.57-7.53) | 6.02(5.04-7.19)^b^ | 7.08(6.36-8.12) | 7.11(6.67-8.86) | 7.33(6.12-7.88) | 6.02(5.07-6.72)^d^ |
| E_2_(pg/mL) | 51(42.25-74) | 50.5(34.75-71.75) | 0.632 | 53(43-78) | 46(38.5-63) | 43.08(33-51) | 57(38-80) | 59.5(46.75-79.5) | 43(35.25-51.75) | 51(36.5-72.5) | 50(34-71) |
| FT3(pmol/L) | 4.57(4.16-4.83) | 4.9(4.57-5.13) | 0.001 | 4.62(4.13-4.88) | 4.56(4.22-4.78) | 4.9(4.43-5.36) | 4.9(4.68-5.07) | 4.69(4.15-5.08) | 4.41(4.14-4.59) | 4.96(4.49-5.21) | 4.89(4.59-5.08)^d^ |
| FT4(pmol/L) | 13.07(12.08-13.89) | 12.95(11.73-13.79) | 0.49 | 13.27(11.94-13.86) | 12.65(12.04-13.78) | 12.93(11.72-13.38) | 12.96(11.77-13.98) | 13.39(12.08-13.9) | 12.65(12.04-13.94) | 13.38(12.9-13.9) | 12.53(11.6-13.83) |
| TSH(uIU/mL) | 2(1.39-2.29) | 1.68(1.15-2.47) | 0.512 | 2(1.42-2.3) | 2.04(1.35-2.23) | 1.3(0.97-1.9) | 2.1(1.19-2.81) | 2.01(1.41-2.29) | 1.98(1.26-2.28) | 1.43(0.9-2.5) | 1.74(1.24-2.48) |
| FINS(mU/L) | 8(6.85-11.45) | 13.9(9.1-23) | <0.001 | 7.55(6.18-8.83) | 14(11.7-16.92) | 8.1(6.5-9.3) | 20.2(13.83-25.08) | 7.5(6.05-8.75) | 10.85(9.03-15.43) | 8.1(6.45-11.2) | 19.5(11.43-23.68) |
| AMH(ng/mL） | 3.05(1.99-4.62) | 8.04(5.35-11.6) | <0.001 | 2.82(2.09-7) | 3.29(1.56-4.04) | 8.37(4.48-14.09)^a^ | 7.86(5.42-10.63)^b^ | 2.58(2.12-3.7) | 4.28(1.35-6.64) | 8.37(6.1-13.79)^c^ | 7.95(4.63-10.72)^d^ |
| HOMA-IR | 1.93(1.61-2.59) | 3.49(2.03-5.67) | <0.001 | 1.71(1.39-2) | 3.35(2.73-3.77) | 1.81(1.38-2.15) | 4.81(3.48-6.38) | 1.68(1.37-2.07) | 2.49(2.02-3.53) | 1.84(1.33-2.55) | 4.19(2.88-6.3) |

Data are presented as Median (IQR).

Abbreviations: IR, insulin resistance; NIR, not insulin resistance; OW, overweight; PCOS, polycystic ovary syndrome; NOW, not overweight; WHR, waist-to-hip ratio; TP, total protein; ALB, albumin; A/G, albumin/globulin; AST, aspartate aminotransferase; ALT, alanine aminotransferase; GGT, γ-glutamyltransferase; ALP, alkaline phosphatase; PALB, prealbumin; CHE, cholinesterase; TBIL, total bilirubin; DBIL, direct bilirubin; IDBIL, indirect bilirubin; TBA, total bile acid; MAO, monoamine oxidase; CHOL, total cholesterol; TG, triglycerides; HDL-C, high density lipoprotein cholesterol; LDL-C, low density lipoprotein cholesterol; apoA1, apolipoprotein A1; apoB, apolipoprotein B; sd-LDL, small dense low-density lipoprotein–cholesterol; FPG, fasting plasma glucose; 25-(OH)VitD, 25-hydroxy vitamin D determination; Prog, progesterone; PRL, prolactin; SHBG, sex hormone–binding globulin; FAI, free androgen index; TT, total testosterone; LH, luteinizing hormone; FSH, follicle-stimulating hormone; E_2_, estradiol; FT3, free triiodothyronine 3; FT4, free triiodothyronine 4; TSH, thyroid stimulating hormone; FINS, fasting plasma insulin; AMH, anti-Müllerian hormone; HOMA-IR, homeostasis model assessment.

^a^ PCOS-NIR vs. control-NIR, *P* < 0.05.

^b^ PCOS-IR vs. control-NIR, *P* < 0.05.

^c^ PCOS-UOW vs. control-UOW, *P* < 0.05.

^d^ PCOS-OW vs. control-OW, *P* < 0.05.
